# V-Shaped Tröger Oligothiophenes Boost Triplet Formation by CT Mediation and Symmetry Breaking

**DOI:** 10.1021/jacs.3c06916

**Published:** 2023-12-07

**Authors:** Samara Medina Rivero, Matías J. Alonso-Navarro, Claire Tonnelé, Jose M. Marín-Beloqui, Fátima Suárez-Blas, Tracey M. Clarke, Seongsoo Kang, Juwon Oh, M. Mar Ramos, Dongho Kim, David Casanova, José L. Segura, Juan Casado

**Affiliations:** † Department of Physical Chemistry, Faculty of Science, University of Málaga, 29071 Málaga, Spain; ‡ Department of Physics and Astronomy, University of Sheffield, Sheffield S3 7RH, United Kingdom; § Organic Chemistry Department, Faculty of Chemistry, Complutense University of Madrid, 28040 Madrid, Spain; ∥ Chemical and Environmental Technology Department, Rey Juan Carlos University, 28933 Madrid, Spain; ⊥ Donostia International Physics Center (DIPC), 20018 Donostia, Euskadi, Spain; # Department of Chemistry, University College London, London WC1H 0AJ, U.K.; ∇ Department of Chemistry, Yonsei University, Seoul 03722, Korea; ○ Department of Chemistry, Soonchunhyang University, Asan 31538, Korea; ◆ Division of Energy Materials, Pohang University of Science and Technology (POSTECH), Pohang 37673, Korea; ¶ Ikerbasque Foundation for Science, 48009 Bilbao, Euskadi, Spain

## Abstract

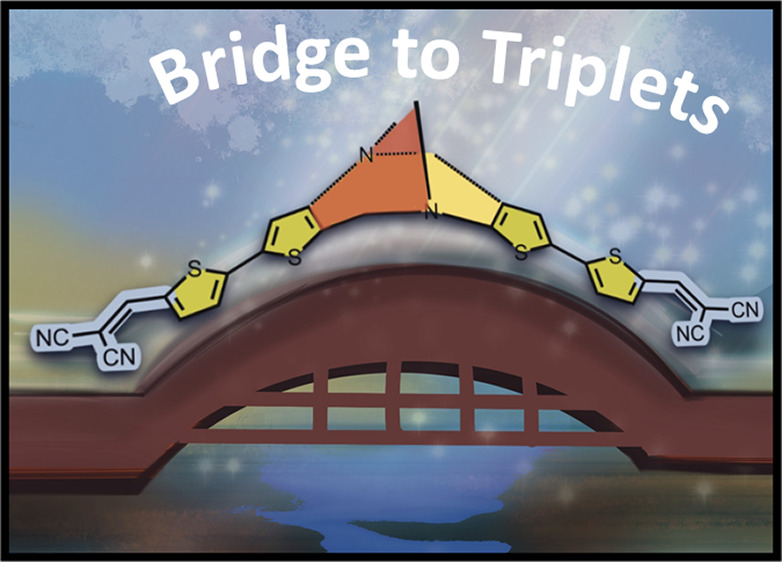

A new family of molecules obtained by coupling Tröger’s base unit with dicyanovinylene-terminated oligothiophenes of different lengths has been synthesized and characterized by steady-state stationary and transient time-resolved spectroscopies. Quantum chemical calculations allow us to interpret and recognize the properties of the stationary excited states as well as the time-dependent mechanisms of singlet-to-triplet coupling. The presence of the diazocine unit in Tröger’s base derivatives is key to efficiently producing singlet-to-triplet intersystem crossing mediated by the role of the nitrogen atoms and of the almost orthogonal disposition of the two thiophene arms. Spin–orbit coupling-mediated interstate intersystem crossing (ISC) is activated by a symmetry-breaking process in the first singlet excited state with partial charge transfer character. This mechanism is a characteristic of these molecular triads since the independent dicyanovinylene-oligothiophene branches do not display appreciable ISC. These results show how Tröger’s base coupling of organic chromophores can be used to improve the ISC efficiency and tune their photophysics.

## Introduction

Conjugation in several forms has been extensively documented in the literature.^1,2^ Commonly, π-conjugated molecules are those holding contiguous arrays of p_*z*_ atom orbitals with direct lateral π-overlap, yielding extensive delocalization in the electronic wave functions that are associated with unique electronic properties potentially suitable for a variety of optoelectronic applications.^3−5^ Photonics is one of the pivotal fields of optoelectronic applications of π-delocalized conjugated molecules in which the relevant photophysical processes are dictated by the nature and strength of electronic conjugative interactions. Excited state energy alignments and interstate couplings tune the competition between energy decay channels, either promoting radiative or nonradiative processes. The variety of available photoinduced processes is especially rich in multichromophoric systems, e.g., in molecular aggregates or covalent dimers and oligomers, in which exciton localization/delocalization and charge separation/recombination play decisive roles. The enhanced versatility and widespread applications of multichromophoric molecules in organic electronics have motivated interest in their design and synthesis. In parallel to their preparation, a comprehensive understanding of the photophysical processes making them suitable for photonics is mandatory for their successful development. In recent years, we have been exploring a variety of conjugation strategies to modulate the electronic properties of π-molecules, such as through-space interactions,^6^ spiroconjugation,^7^ and hyperconjugation,^8^ in order to fine-tune the coupling between excited states produced by the assembly of two or more chromophores.

**Scheme 1:**
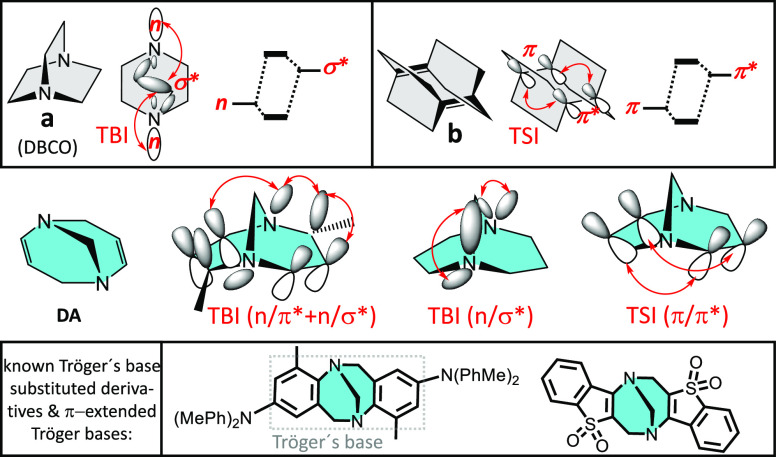
Structures of the **a** and **b** Moieties and That of Diazocine (DA) (i.e., the Bisphenyl-Fused DA Compound Is Known as Tröger’s Base Highlighted in the Dotted Line Box) In **a**, **b**, and DA, orbital representations of TBI and TSI (red arrows) together with their first-order perturbation couplings are shown. Some known examples of Tröger’s base derivatives and bisarene Tröger-based chromophores are shown in the solid line box. Note that the signs of the p_*z*_ orbitals are just to qualitatively describe the interaction (i.e., they do not follow a nodal pattern).

Going to particular examples of the previously mentioned electronic effects, the DBCO fragment (i.e., **a** in Scheme 1 or 1,4-diazabicyclo[2.2.2]octane) represents a typical case with through-bond coupling or interaction (TBI) between the lone electron pairs of the nitrogens (n) assisted by the antibonding sigma orbitals (σ*) of the bismethylene bridge (n/σ* TBI and orbital structure in Scheme 1).^9^ Another example is fragment **b** in Scheme 1, i.e., DBCO with the two nitrogen atoms replaced by vinylenes, where through-space interaction (TSI) occurs between the π orbitals of the two double bonds (π/π* TSI and orbital structure in Scheme 1).^9^ Finally, in the diazocine (DA) ring in Scheme 1, the interactions can be considered as a mixture of those in fragments **a** and **b** (i.e., n/π* and n/σ* TBI between the nitrogens and the vinylenes, n/σ* TBI between the nitrogens, and π/π* TSI between the vinylenes). Extending the DA unit by fusing a benzene ring at each vinylene results in the so-called Tröger’s base (Scheme 1).^10^ The relative energy disposition and symmetry between the interacting orbitals of the DA unit in Tröger’s base define its emergent photophysical behavior, which will be explored here for oligothiophene derivatives. Many π-bonded conjugated derivatives of Tröger’s base have been prepared and used in different ways in chemistry.^11−14^Scheme 1 shows some of these derivatives studied in the context of our investigations.

**Scheme 2:**
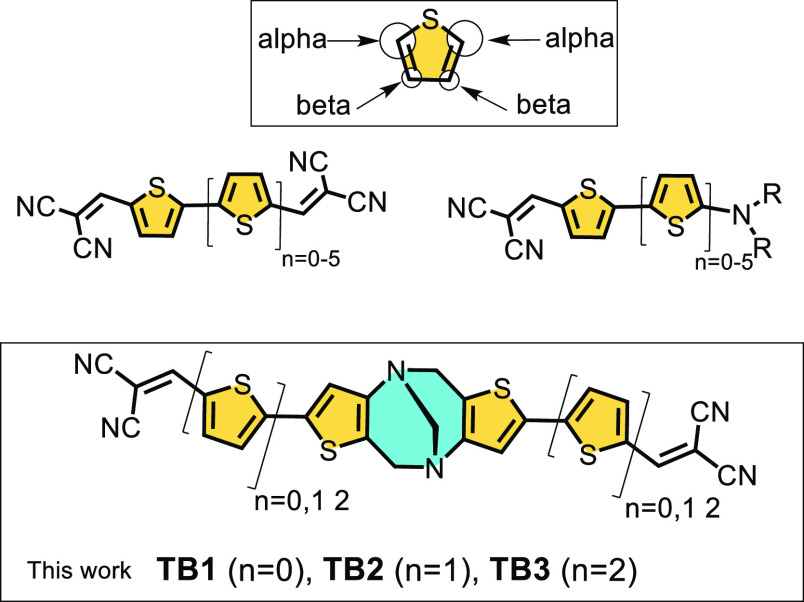
Chemical Structure of Some Symmetrically Dicyanovinylene-Substituted Oligothiophenes (DCVTn) as Well as Donor–Acceptor Dicyanovinylenes with Substitution Pattern through the α Positions of the Thiophenes The chemical structures of the bis(dicyanovinylene) oligothiophenes segmented by the DA unit or thiophenic Tröger’s bases are also shown together with the nomenclature used in this investigation.

Oligothiophenes have been exploited in organic electronics from the very beginning of the research on organic π-conjugated molecules.^15,16^ In the area of photonics, and, in particular, in photovoltaic applications, oligothiophenes have been developed by functionalization of the terminal α,α positions (those contiguous to the sulfur atom) with dicyanovinylene groups (DCVTn in Scheme 2) in order to establish a donor–acceptor π-electronic coupling by which the optical gap and the energy of the relevant molecular orbitals can be tuned.^17−19^ This suitable modulation of the electronic properties is possible due to the large donor–acceptor interaction through the α carbon atoms. On the other hand, substitution with active groups in the β positions of the thiophene moiety (Scheme 2) has been much less studied due to the induced faint electronic effect associated with a lesser electron delocalization. But, in some cases, weak interchromophore interactions might be desirable in applications, for example, in singlet exciton fission.^20^

Our starting hypothesis is that the joint assembly of donor–acceptor thiophenes with Tröger’s base produces multichromophoric systems with multiple modes of interactions mediated either by TBI or TSI mechanisms, conferring either large or weak couplings. In this article, we report the synthesis of new oligothiophene derivatives with different number of thiophenes (mono-, bi-, and terthiophene) functionalized with terminal dicyanovinylene groups and covalently connected by Tröger’s base DA unit, namely, the triads: bis(dicyanovinylene) terminated thiophene (**TB1**), bithiophene (**TB2**), and terthiophene (**TB3**) in Scheme 2. Their steady-state and time-resolved spectroscopic properties have been analyzed, and the main photophysical mechanisms have been described and quantitatively evaluated by means of electronic structure calculations.

## Experimental and Characterization Methods

### Computational Details

Molecular ground and excited state geometries were optimized within the framework of density functional theory (DFT) at the CAM-B3LYP/6-31G(d,p) level. Vertical excitation energies have been obtained with the same exchange-correlation functional and the 6-311+G(d,p) basis set within time-dependent DFT (TDDFT). Results from calculations performed resorting to the Tamm-Dancoff approximation (TDA)^21^ can be found in the SI. Solvation effects have been taken into account by means of polarizable continuum models (PCM) with the integral equation formalism (IEF-PCM)^22,23^ implementation for ground state geometry optimizations and the conductor-like model (C-PCM)^24,25^ for excited state calculations. Quantification of the charge transfer character of electronic excitations has been performed with the TheoDORE program.^26^ All calculations were done using Gaussian 16^27^ and Q-Chem^28^ program packages.

### Electronic Absorption and Emission Spectroscopy

Room-temperature UV–vis absorption spectra were recorded on a Varian Cary 5000 UV–vis-NIR spectrophotometer operating in the range of 175–3300 nm using a PbSmart NIR detector, while room-temperature emission spectra were obtained using an FLS920P spectrofluorometer from Edinburgh Analytical equipped with a 400 W continuous wavelength Xenon lamp, an nF920 ultrafast nanosecond flashlamp, and an nF900 ns pulsed flashlamp as well as a range of picosecond pulsed laser diodes (EPL Series: EPL375 (λ = 374.6 nm), EPL405 (λ = 404.8 nm), and EPL475 (λ = 472 nm)), and nanosecond pulsed LEDs from PicoQuant (PLS-λ Series: λ = 280, 300, 340, 370, 450, 500, and 600 nm). Detection can be performed in the 200–800 nm (R928P) or 200–1000 nm (R2658P) spectral ranges. Variable temperature absorption and emission spectra were obtained with an Optistat DN Oxford Instruments cryostat, which allows sample temperature variations from −196 to 200 °C. For this purpose, the employed solvent was 2-methyl-tetrahydrofuran (2-MeTHF, Sigma- Aldrich/Merck, Anhydrous, ≥ 99%) since it provides a transparent frozen matrix at low temperatures. Titration experiments were conducted in dichloromethane at room temperature by progressive addition of trifluoroacetic acid (TFA; Sigma-Aldrich, reagent plus, 99%, CAS: 76-05-1) to a 10^–6^ M solution of the corresponding **TBn** oligomer.

### Picosecond Transient Absorption Spectroscopy

Pump and probe beams were generated using a Ti:sapphire regenerative amplifier (Spitfire Ace, Spectra-Physics) providing 800 nm pulses (120 fs full-width at half-maximum-fwhm, ≤ 1 kHz repetition rate, average power of 5W). Spitfire Ace is operated with a Mai Tai Ti:Sapphire seed laser (tunable range: 690–1040 nm, 100 fs fwhm, 80 MHz, 3W, Spectra-Physics) and a Nd:YLF Empower 45 Q-switched pump laser (527 nm, 10 ns fwhm, 1 kHz, 15W, Spectra-Physics). Tunable narrowband pump pulses at 415, 450, and 500 nm for **TB1**, **TB2**, and **TB3**, respectively, were generated in a TOPAS Prime optical parametric amplifier (output tunable range: 290–1600 nm, Spectra-Physics) with an output power of 1 mW. Probe pulses spanning the 350–700 nm range were generated by focusing a portion of the 800 nm beam through a continuously translating calcium fluoride crystal. Pump–probe delay was controlled using a direct-drive high-speed optical delay line with a standard 8 ns time window. Detection was carried out using a Helios automated femtosecond transient absorption spectrometer equipped with CMOS (UV–vis: 350–950 nm, spectral resolution: 1.5 nm) and InGaAs (NIR: 800–1600 nm, spectral resolution: 3.5 nm) detectors. Each spectrum corresponds to an average of 3 scans.

### Microsecond Transient Absorption Spectroscopy

Microsecond TAS was recorded by using a 6 ns, 10 Hz Nd:YAG laser (Spectra-Physics, INDI-40-10) for the excitation pulse. The excitation wavelength was selected with a versaScan L-532 OPO. Excitation density was set from 3 to 200 μJ/cm^2^ using neutral density filters and measured with an ES111C sensor (Thorlabs). Probe light was provided by a quartz tungsten halogen lamp (IL1, Bentham). The TA signals were recorded with Si and InGaAs photodiodes coupled to a preamplifier and an electronic filter (Costronic Electronics) connected to a Tektronix DPO4034B oscilloscope and PC. Probe wavelengths were selected with a Cornerstone 130 monochromator (Oriel Instruments) before the detector. To measure the oxygen atmosphere decays, pure oxygen gas was bubbled directly into the solution to dissolve oxygen. After that, freeze–pump–thaw was performed to remove all oxygen to check for TA signal recovery.

## Results and Discussion

### Synthesis

**Scheme 3:**
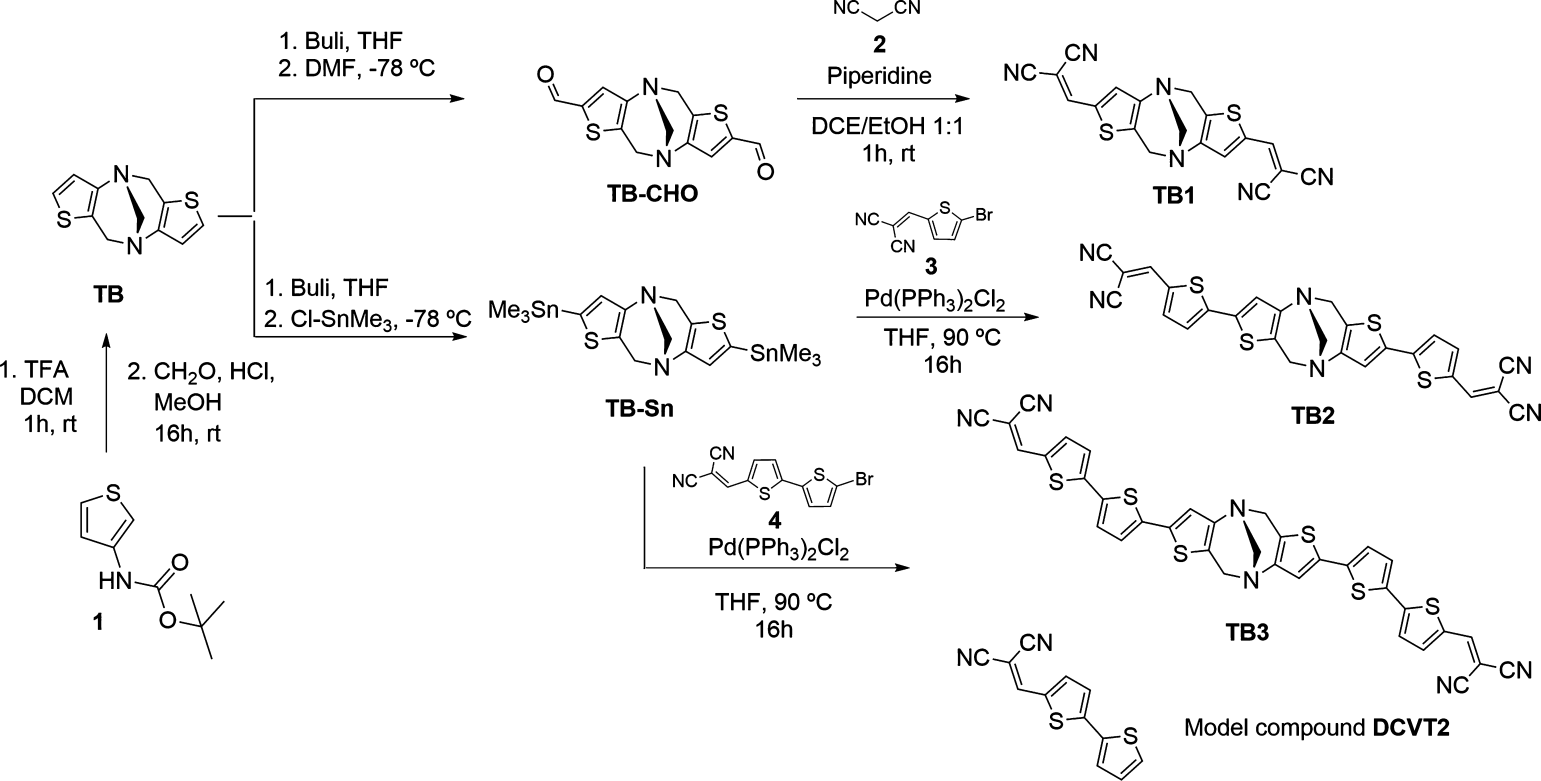
Syntheses of the New Oligothiophene Congeners of Tröger’s Base Analogues **TB1, TB2**, and **TB3** and the Model Compound **DCVT2**

The preparation of donor–acceptor (D-A) assemblies derived from the thiophene congener of Tröger’s base (**TB**) is described in Scheme 3. The synthetic route starts from the commercially available β-amino protected thiophene, which is treated in acid media to obtain the unstable β-amino thiophene^29^ that is in turn directly used to obtain **TB** in a similar way to that described by Kobayashi and co-workers.^30^ The treatment of **TB** with butyl lithium in the presence of an electrophilic reagent such as N,N-dimethylformamide or trimethyltin chloride led to the obtention of the precursors **TB-CHO** and **TB-Sn** of the target molecules. Finally, a Knoevenagel-like reaction^31,32^ between the formylated **TB-CHO** with malononitrile **2** gives **TB1** derivative under mild reaction conditions. To obtain **TB2** and **TB3** assemblies, Stille cross-coupling reactions between the stannyl derivative **TB-Sn** and the corresponding dibromo thiophenes **3** and **4** were carried out in good yields. The chemical structures for these four novel compounds (**TB** and **TBn**) have been properly characterized by mass spectrometry, FT-IR, and nuclear magnetic resonance techniques (Figures S1–S15). The **DCVT2** compound—representing the monomer control for comparison to Tröger’s base dimer, **TB2**—in the discussion corresponds to the nonbrominated version of **4** in Scheme 3.

### Electronic and Photophysical Properties of Tröger-Spaced Oligothiophenes

#### Coupling of Dicyanovinylene-Oligothiophenes through the DA Linker

The properties of **TB1–3** molecules can be rationalized as emerging from the coupling of two dicyanovinylene-oligothiophene fragments through Tröger’s base DA core. The rigid V-shape of the DA central linker imposes a nearly perpendicular and slightly twisted arrangement of the two DCVTn moieties, as illustrated by the ground state equilibrium geometry obtained for **TB1** (Figure S16 and Table S1).

The most relevant structural parameters for all compounds are reported in Table S1. It is worth noting that two quasi-isoenergetic (Δ*G* < 1 kcal mol^–1^) structural conformers were found for each molecule, related to the *cis*/*trans* isomerism of the vinylene of the dicyanovinylene moiety with respect to the double bond of the thiophene (Table S2). Unless indicated, computational results presented in the following correspond to the *cis* isomers (results for the *trans* isomers can be found at the end of the ESI file).

**Figure 1:**
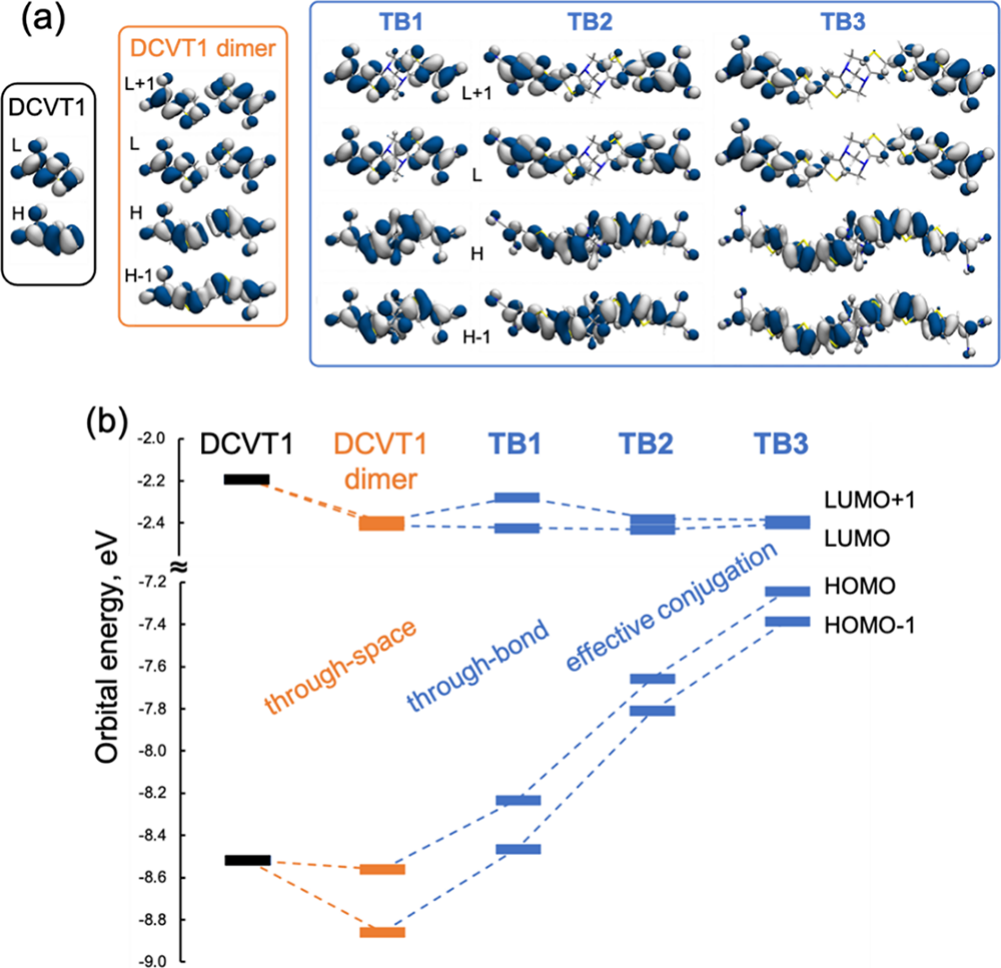
Frontier molecular orbitals (a) and their energy diagram (b) for the **DCVT1** moiety (black), **DCVT1** dimer (orange) and **TB1**, **TB2**, and **TB3** compounds (blue), computed at the CAM-B3LYP/6-311+G(d,p) level. H: HOMO, L: LUMO.

Frontier molecular orbitals of **TB1** are symmetrically delocalized around the DA center and mostly correspond to in-phase and out-of-phase combinations of **DCVT1**’s HOMO and LUMO (Figure 1a). Whereas the HOMO and HOMO–1 are delocalized over the whole molecule, the LUMO and LUMO+1 only have contributions to the **DCVT1** fragments, suggesting possible low-energy charge transfer channels from the central donor to the acceptor chromophores. Molecular orbitals of **TB2** and **TB3** follow equivalent profiles with HOMOs fully delocalized and LUMOs symmetrically arranged at the two DCVTn branches.

In order to characterize the electronic role of the DA fragment, we compare orbital energies of **TBn** molecules with those of the **DCVT1** unit and that of a **DCVT1** noncovalent dimer, or simply **DCVT1** dimer, with the two **DCVT1** units spatially arranged as in **TB1** (Figure 1). TSIs in the **DCVT1** dimer stabilize its HOMO–1 bond (with respect to the HOMO of the **DCVT1** unit), resulting in a sizable HOMO/HOMO–1 gap. On the other hand, the LUMO and LUMO+1 of the noncovalent **DCVT1** dimer are stabilized relative to those of the single **DCVT1** unit and remain nearly degenerate. In the presence of the DA bridge, TBIs contribute to increasing the effective conjugation of the π-occupied orbitals, progressively destabilizing the two HOMOs with the size of the molecule, i.e., from **TB1** to **TB3**, while the energy of the LUMOs remains nearly unaffected.

Overall, this description reveals that the presence of the DA bridge promotes: (i) TBIs contributing to increase the effective conjugation on the π-occupied orbitals of the triads (i.e., changes from the noncovalent **DCVT1** dimer to **TB1**), as well as progressively destabilizing the two HOMOs with the size of the molecule (i.e., from **TB1** to **TB3**); and (ii) the lowering of the energy of the π LUMOs by through-space dimerization and TSI (i.e., changes from **DCVT1** to the **DCVT1** dimer), while these are only weakly affected from the **DCVT1** dimer to **TB1**, in accordance with the negligible participation of the DA unit in the LUMOs (Figure 1).

#### Low-Lying Singlet Excited States and Electronic Absorption Spectra

**Figure 2:**
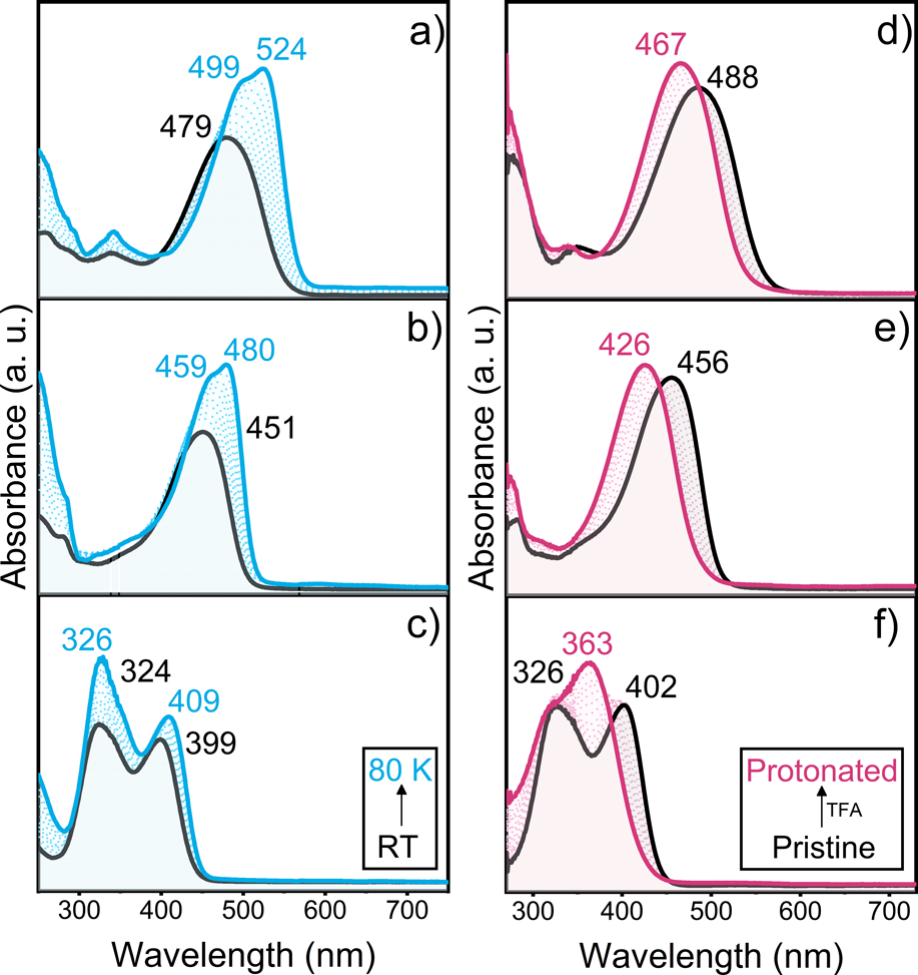
UV–vis electronic absorption spectra of **TB3** (panels a and d), **TB2** (panels b and e), and **TB1** (panels c and f) at room temperature (black lines) and at 80 K (blue lines) in 2-MeTHF (left) and at room temperature (black lines) upon treatment with TFA (pink lines) in CH_2_Cl_2_ solution (right).

The frontier orbital distribution of **TBn** molecules (Figure S17) defines the properties of the low-energy electronic transitions. TDDFT calculations indicate that the first excited state (S_1_) in **TB1–3** is dominated by HOMO-to-LUMO and, to a lesser degree, HOMO–1-to-LUMO+1 one-electron promotions (Table S3). The second excited state (S_2_) is described as a linear combination of HOMO–1→LUMO and HOMO→LUMO+1 excitations. Both transitions present a rather strong oscillator strength, especially the S_1_ one, which allows one to associate them with the low-energy broad absorption bands measured in 2Me-THF solution at room temperature (Figure 2).

Increasing the number of thiophene units results in a redshift of the absorption wavelength maxima of the experimental spectra. This spectral displacement is well reproduced by electronic structure calculations (Table S3). Cooling the solutions of the three compounds up to 80 K produces a net redshift of the absorption bands compared to the spectra at 298 K and the appearance of vibronic structure due to the collapse of low-frequency modes, such as the inter-ring torsional vibrational modes, on removing thermal energy. As a result, the vibronic resolution is more evident in the two longer compounds, where interthiophene flexibility is present.

**Figure 3:**
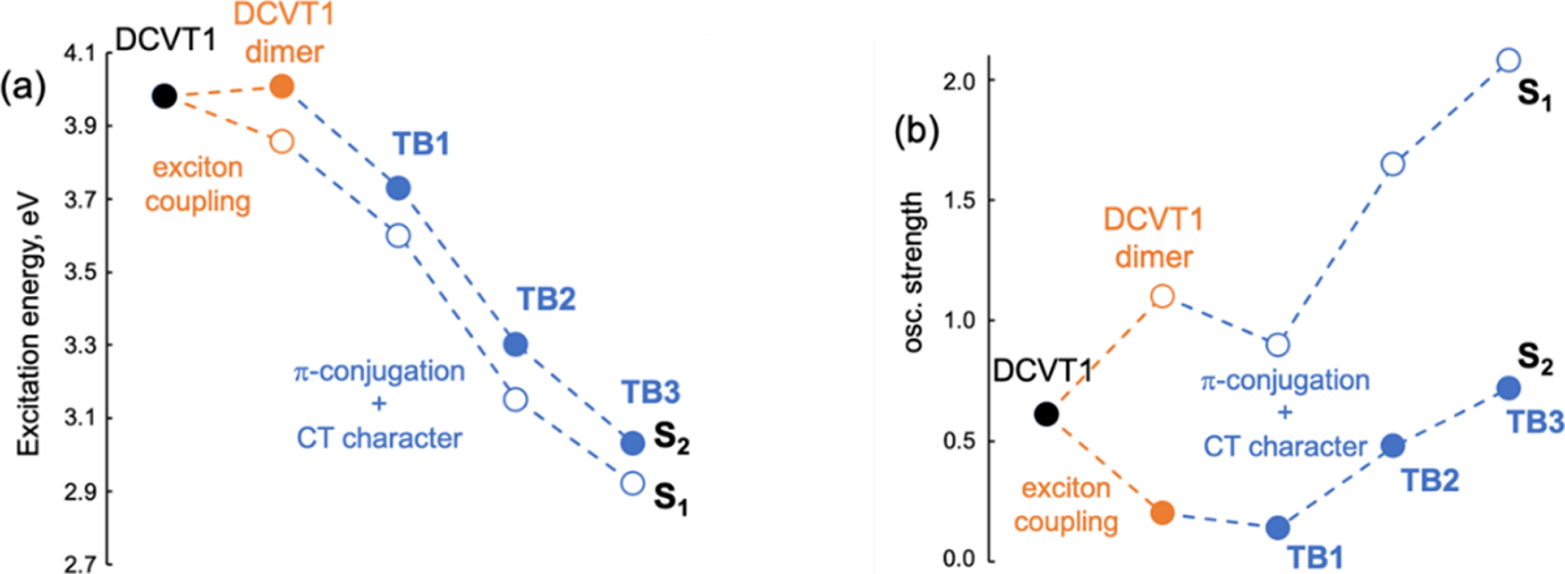
Vertical excitation energies (a) and their oscillator strengths (b) for the S_1_ (empty circles) and S_2_ (filled circles) transitions for the following systems: the **DCVT1** moiety (black) and its through-space dimer, DCVT dimer (orange), and the target molecules **TB1**, **TB2**, and **TB3** compounds (blue). Quantum chemical calculations are performed at the CAM-B3LYP/6-311+G(d,p) level.

To characterize the nature of these transitions, we rely again on the comparison with the calculated **DCVT1** monomer and the noncovalent **DCVT1** dimer. Exciton coupling between local excitations in the **DCVT1** dimer (computed at the ground state geometry) splits the energy and oscillator strength of the transition to S_1_ and S_2_ with respect to the **DCVT1** monomer computed values (Figure 3). These quantum chemical excitation energies and transition dipole moment values are in good agreement with those obtained using a classical dipole–dipole interaction (Figure S18). Covalent linkage of the two **DCVT1** units through the DA bridge in **TB1** reduces the excitation energies of both singlets, in agreement with the (theoretical and experimental) wavelength redshifts of the absorption maxima, which can be related to the extension of π-conjugation that induces destabilization of the HOMOs as well as the anticipated DA→**DCVT1** CT character of the orbital transitions (Figure 1). As a consequence of the CT mixing, oscillator strengths in **TB1** decrease with respect to those in the **DCVT1** dimer (Figure 3b). Conversely, the oscillator strength of the S_1_ and S_2_ pair of transitions increases with the size of the molecule due to the large overlap between occupied and virtual orbitals and the concomitant increase of the hole/electron separation along the series. The comparison between **DCVT2** and **TB2** can be found in Table S3, with similar results.

#### Composition of the Excited States of **TBn**

We aim to evaluate whether indeed the transition to S_1_ has some CT character. For that, we divide the **TBn** triads into three moieties, i.e., chromophore-bridge-chromophore, and perform a fragment-based analysis of the one-electron transition density matrix as implemented in the TheoDORE program.^26^ Since there is not a unique way to define the three fragments, we explore different possible partitions by defining the central fragment as the DA or **TB** (DA + 2 thiophenes), or the side chromophores as **DCVT1** (1 thiophene ring) (Figure S20). Of course, the results, i.e., the amount of CT contributions, change with the chosen partition, with CT character values reaching the 20–50% range in most of the cases. Despite the quantitative differences upon the chosen partition, these results univocally characterize S_1_ as the coupling of local excitations on each DCVTn unit with some appreciable CT character from the central linker to the side chromophores. Moreover, the analysis at the atomistic level identifies CT terms arising mainly from the nitrogen atoms in the DA unit. Further details of these results can be found in the Supporting Information.

The participation of the DA unit, in particular its N atoms, as an electron donor moiety in the low-lying transitions is further demonstrated by the changes in the photophysical properties upon protonation. The electronic absorption spectra of the three compounds upon treatment with TFA are displayed in Figure 2. Protonation of the nitrogen atoms of DA blue-shifts the absorption maximum of all compounds with respect to those of the pristine molecules, with the magnitude of the spectral displacement decreasing with the size of the system (0.33 eV for **TB1**, 0.19 eV for **TB2** and 0.11 eV for **TB3**). The molecular orbitals involved in the transition to the low-lying excited states are strongly stabilized in the protonated form of **TB1**, with a larger effect on the occupied ones (HOMO and HOMO–1, which presented significant contribution over the nitrogen atoms of the DA moiety in **TB1**), resulting in an overall blue-shift of the bands. In the absence of the nitrogen’s lone pair on the central unit, the CT character is severely reduced and S_1_ is now characterized as a symmetric combination of local excitations (Figure S19).

Therefore, we conclude that the S_1_ (and S_2_) state in **TBn** is mostly obtained as the exciton coupling between local excitations on DCVTn fragments with partial (DA→DCVTn) CT contributions showing major participation of the nitrogen’s electron lone pairs. These results demonstrate the dual conformational and electronic role of the DA moiety in the low-lying excited states of **TB1–3**: (i) first, it plays a conformational/structural role in bringing the two DCVTn fragments in a quasi-perpendicular arrangement with a short distance at the thiophene sides (about 3 Å), and (ii) it participates in the electronic coupling of the two units via CT contributions mainly involving the lone pairs of the two nitrogen atoms. Overall, this scenario might reveal an excited state symmetry-breaking mechanism acting together with a CT effect in S_1_ after photon excitation.

#### Fluorescence

**Figure 4:**
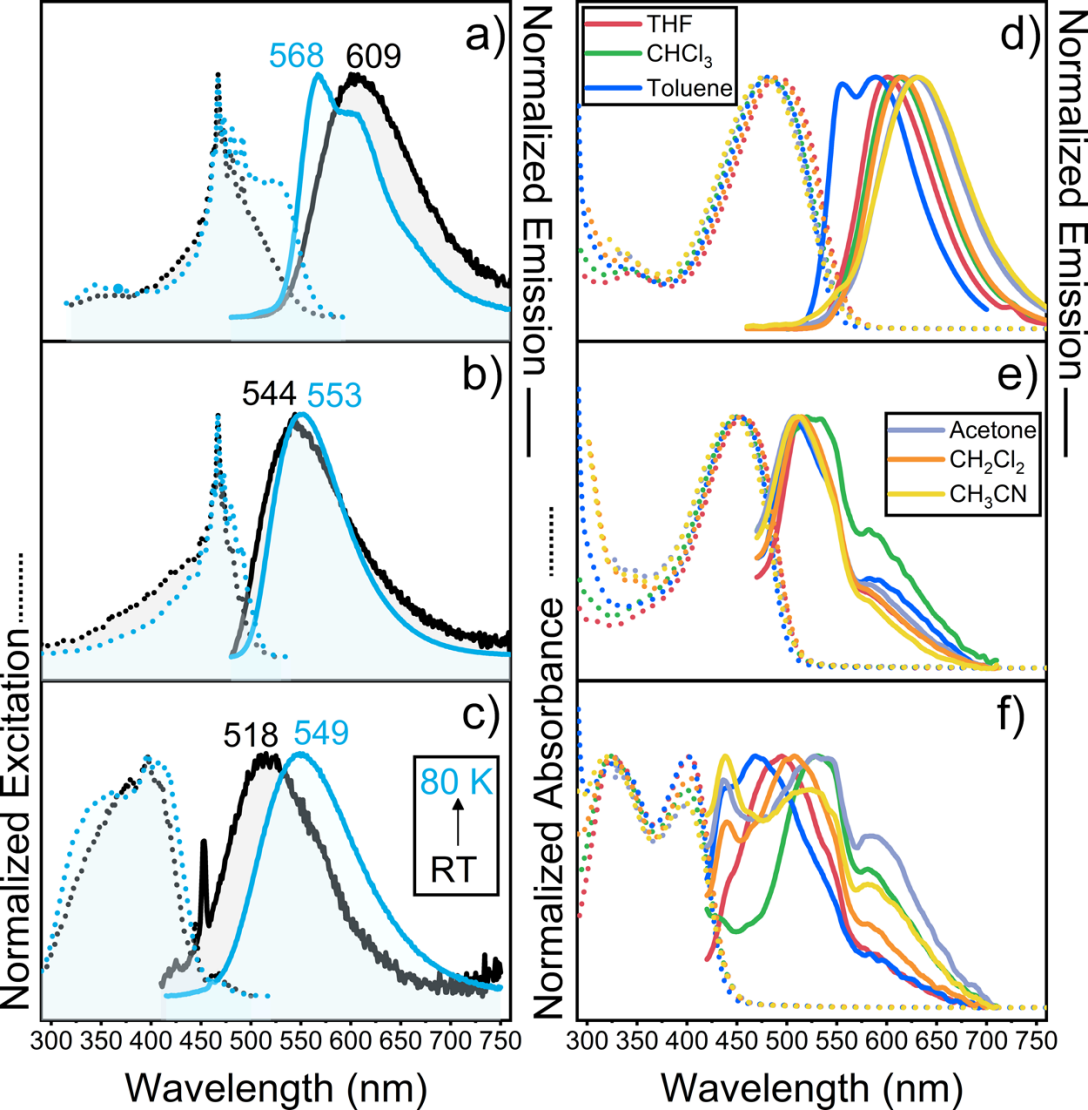
Left: excitation (dotted lines) and emission (solid lines) of **TBn** at room temperature (black lines) and at 80 K (blue lines) in 2-MeTHF. Right: absorption (dotted lines) and emission (solid lines) spectra of **TBn** at room temperature in toluene (dark blue lines), CHCl_3_ (red lines), THF (green lines), CH_2_Cl_2_ (orange lines), acetone (gray lines), and CH_3_CN (yellow lines). From top to bottom: (a and d) **TB3**, (b and e) **TB2**, and (c and f) **TB1**. Sharp peaks at ca. 450–470 nm in panels (b) and (c), which are not present in the absorption spectra, are ascribed to scattering artifacts.

The excitation and emission spectra of the three compounds in toluene at room temperature shown in Figure 4 present in all cases moderate Stokes shifts and fluorescence quantum yields that are <1% for **TB1** and **TB2**, and 6.6% in **TB3**, in line with the small radiative decay lifetime of 0.16 ns for **TB3** in toluene (lifetimes for **TB1** and **TB2** could not be measured). These emission spectra for each molecule are all identical irrespective of the excitation wavelength.

Noticeably, the absorption spectra in THF (a more polar solvent) were identical to those in toluene. However, the emission bands display clear differences at higher solvent polarity: (i) they are significantly redshifted compared to toluene and (ii) the fluorescence quantum yields, despite the redshifts, are higher in THF, with that of **TB3** amounting to 19.1%. These solvatochromic behaviors of the **TBn** series clearly describe their photoinduced CT nature, where the more polar solvent leads to a larger stabilization of the CT state, and CT states are substantially less fluorescent than local excitations. Moreover, the solvent-polarity dependence of the fluorescent features of **TB3** differs from those of **TB1** and **TB2**, both in fluorescence quantum yields and in band shapes.

These contrasting emission characteristics are more marked at a low temperature, 80 K. By cooling, **TB1** and **TB2** show redshifted fluorescent emission bands compared to 298 K, whereas that of **TB3** is blue-shifted. This suggests that the electronic nature of the emitting state of the **TBn** compounds is altered upon increasing the number of thiophene units, and that the distinct emission behavior of **TBn** can be accounted for by the conjugative effect along the DCVTn units. As shown in Figure 1, the (partial) CT nature of the **TBn** excited states is well illustrated by the spatial distribution of their frontier molecular orbitals. Taking a close look at their LUMO and LUMO+1, the electron density on the central DA and fused thiophenes is significantly reduced from **TB1** to **TB2** and to **TB3**. In particular, the electron density is mostly concentrated over the external thiophenes and the dicyanovinylene moieties, with only a very minor contribution over the central DA unit. This conjugative effect toward the terminal molecular parts is well represented by the blue-shifted fluorescence of **TB3** at 80 K. Typically, oligothiophenes show dynamical planarization processes in the excited state by enhancing the conjugative delocalization and consequently reduce the dihedral angles between thiophenes, or torsional relaxation.^33^ Thus, under frozen conditions, the torsional relaxation of **DCVT3** in **TB3** might be blocked and emission occurs from a high-energy vibronic state of S_1_, resulting in a blue-shifted emission at 80 K. Since **DCVT1** in **TB1** only has one thiophene ring per side, interthiophene planarization in S_1_ is not present, resulting in redshifted emission and an fwhm at 80 K comparable to that observed at 298 K, as a decrease in temperature only leads to an increase in overall molecular rigidity of **TB1** associated with the observed redshifts. **TB2** also shows redshifted spectra upon solvent cooling at 80 K with a decreased fwhm of the emission band. This can be understood as a compensation for spectral changes in **TB2** between **TB1** and **TB3**. Here, it is noteworthy that the correlation between the change in fluorescence features and the increase in thiophene units of **TBn** suggests qualitative differences in the structural relaxation of the excited state.

Absorption of light followed by relaxation on the S_1_ potential energy surface (PES) produces significant geometrical changes within one DCVTn subunit and half of the DA linker in comparison with the ground state geometry. This structural rearrangement is driven by exciton localization on one side of the molecule and involves the symmetry breaking of the CT contributions (Figure S21). For all compounds, the lowest excited state at the PES minima is associated with a HOMO→LUMO electron promotion. In line with the structural rearrangements observed in the relaxed S_1_ equilibrium geometry, the frontier orbitals of the three compounds are localized on half of the molecules (Figure S22). The symmetry-breaking localization of the excited state is further illustrated by fragment-based analysis, which indicates that the relaxed S_1_ state presents a quasi-pure locally excited (LE) character mostly involving one of the DCVTn units (Figures S23 and S24).

Electronic structure calculations recover the experimentally observed trend in all compounds, which can be rationalized due to the increase in the relaxed (localized) excited state permanent dipole moment with the solvent polarity (Table S6). Furthermore, the null dependence of the absorption spectra in contrast with the large solvatochromic variation of the emission ones is in agreement with an excited state symmetry-breaking process, in which upon vertical excitation the delocalized excited state evolves (i.e., by means of coupling with appropriate symmetry CC stretching vibrational modes) toward a more localized state that is increasingly stabilized by solvent polarity (i.e., producing redshifts). Excited state symmetry-breaking processes have been described for other Tröger’s base derivatives.^11,12,14^

#### Excited State Relaxation Dynamics

**Figure 5:**
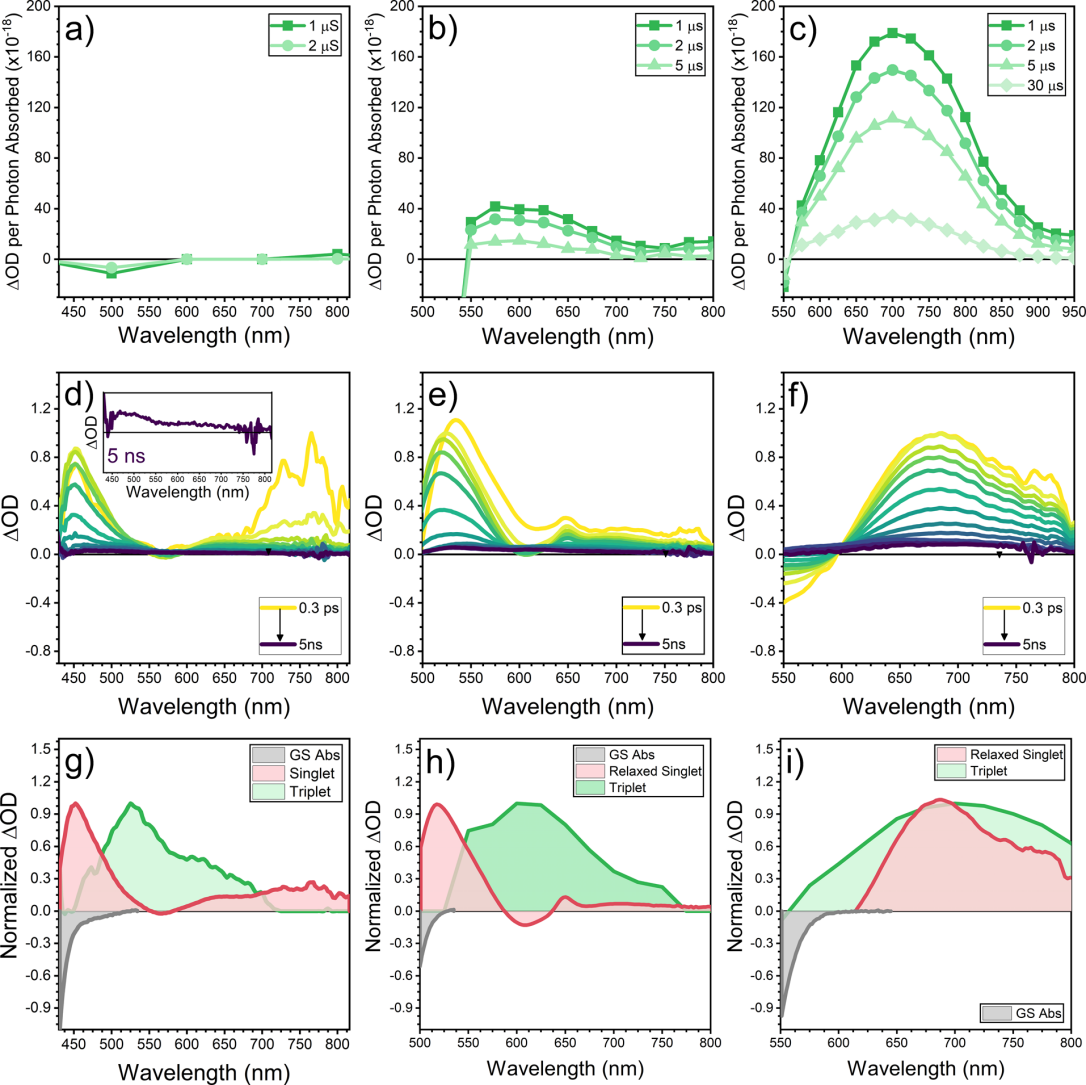
Microsecond (top) and picosecond (middle) time-resolved transient absorption spectra in CH_2_Cl_2_ at 298 K. Excited state spectra comparisons (bottom) from global analysis. (a, d, and g) **TB1**, (b, e, and h) **TB2**, and (c, f, and i) **TB3**. Microsecond TAS was obtained upon excitation at 100 μJ/cm^2^ at 415, 450, and 500 nm for **TB1**, **TB2**, and **TB3**, respectively. ps-TAS was obtained upon excitation at the same wavelength but with a power of 0.5 mW.

Figure 5 (top) shows the microsecond transient electronic absorption spectra (μs-TA) of **TB1**, **TB2**, and **TB3** with the *y*-axis normalized per photon absorbed.

The μs-TA spectrum of **TB1** shows negligible excited state absorbance (ESA) features (Figure 5a). For **TB2**, however, a band in the μs-TA is present at 600 nm (Figure 5b), which decays with a lifetime of 4 μs. **TB3** shows the band with the largest intensity of the three compounds, with a decay lifetime of 50 μs (Figure 5c), a much larger value than those in **TB1** and **TB2** (Figure S25). The reversible quenching of the TA bands with oxygen, later recovered when purged again with nitrogen, unequivocally proves the assignment of these two bands to **TB2** and **TB3** triplets (Figure S26), and consequently, the discussed lifetimes correspond to the decay of long-lived triplet excited species. According to this sequence, in which the triplet lifetime increases from **TB2** to **TB3**, it is reasonable to assume that the triplet lifetime in **TB1** could be smaller than the instrumental resolution. This interpretation is in agreement with the recent report of highly efficient intersystem crossing (ISC) in Tröger’s base derivatives.^14^

To further understand the photoinduced processes in these molecules, we have also carried out picosecond transient absorption (ps-TA) measurements (Figure 5, middle). For **TB1**, photoexcitation at 415 nm leads to the appearance of two main ESA bands at 450 and 750 nm that decay via two distinct routes, with two different lifetimes: 10 and 49 ps. Both lifetimes were independent of the excitation density, which rules out any annihilation process (Figure S27). Interestingly, also at 10 ps, a new ESA band with a peak at 530 nm extending up to 650 nm rises with a lifetime comprised between 100 ps and the instrumental resolution (6 ns). The initially photopumped excited state of **TB2** decays to the ground electronic state with a 37 ps lifetime. The S_1_ → S_0_ deactivation route coexists with the population of an excited state generated in 17 ps and characterized by an ESA spectrum that displays the main broadband with a peak at 600 nm persisting after 5 ns. The high resemblance of this spectrum formed upon 17 ps with the spectrum recorded in the microsecond time regime means that it can be assigned to the formation of triplet excited state species. A similar picosecond excited state dynamics is found for **TB3**, in which hot singlet excited state relaxation occurs after 1.2 ps, followed by the recovery of the ground electronic state in 160 ps (i.e., in line with the fluorescence lifetime for **TB3** of 0.16 ns). In parallel, a partial conversion from this photopumped singlet state takes place within 40 ps, giving rise to a species that survives over the entire resolution time of the instrument. The photoinduced absorption spectrum of this last species is very broad, with a maximum of 700 nm. Moreover, it presents a great resemblance with the photoinduced absorption band in the μs-TA spectrum of **TB3**, suggesting its assignment to the triplet state. This result nicely explains why this excited state with slow relaxation dynamics is not seen to decay in the picosecond-nanosecond interval. The negative broadband in **TB3** arises from stimulated emission with a profile similar to the fluorescence spectrum in Figure 4. In addition, ps-TAS data were obtained for **TB**n in solvents with different polarities (Figure S28). For **TB1** and **TB2** we observe an increase in the excited state lifetime with a decrease in the solvent polarity. This indicates a clear CT character of the excited species, in line with our previous assignment. In the case of **TB3**, the analysis is more complicated given that a more stable CT state could be formed in the more polar THF and the larger thiophene arm (three thiophenes), which might allow a better separation of charge density. As a result, this CT state has a deeper energy level, and therefore, the triplet formation is limited, as seen by the low intensity of the signal upon 6 ns.

From this comparison, it is clear that the formation of triplet species in **TB2** and **TB3** might occur in an ultrafast picosecond process giving rise to a conventional triplet species that decays in microseconds. This very rapid formation of triplets upon photopopulation of the bright singlet excited state is considerably faster than common ISC but consistent with other examples in the literature.^34−36^ By extrapolation of **TB2** and **TB3** ISC rates to **TB1**, we can deduce that the transition of the starting singlet excited state to a triplet excited state occurs within 10 ps, with this species now quickly decaying to the ground electronic state in the nanosecond time scale (i.e., it is not observed in the microsecond time-resolved experiment). Therefore, this process, i.e., intersystem crossing, is progressively slowed down from **TB1** to **TB2** and **TB3**, with ISC transition times of 10, 17, and 40 ps (Figure S29), respectively.

The common difficulties of obtaining an absolute value for triplet absorptivity, besides the small absorbance of the triplet bands in the ps-TAS spectra, preclude an accurate quantification of the triplet yield. In addition, low absolute triplet yields are expected as the formation of CT states that mediate the formation of triplets might also accelerate internal conversion to the ground state. Therefore, we qualitatively address the relative triplet quantum yield among the different **TBn** materials assuming the changes in molar absorptivity from singlet to triplet should be similar from molecule to molecule. Then, with the data obtained by global analysis (GA) of the ps-TAS data (Figure S30) and assuming no triplet has decayed while the triplet population raises, ΔOD_max-S_/ ΔOD_max-T_ should give an estimate of the triplet yields which amount such as follows: 5, 7, and 12% of triplet yield for **TB1**, **TB2,** and **TB3**, respectively.

To further support this discussion, a monomer reference of **TB2** (i.e., **DCVT2** in Figure S31 which is the nonbrominated version of **4** in Scheme 3) has been prepared. Figure S32 shows the excitation and emission spectra of **DCVT2** from which it is inferred how the DA units intervene by producing a redshift of the emission band of **TB2** compared with **DCVT2**. In addition, ps-TAS spectra reveal that all excited species populated in **DCVT2** relax back to the ground state within 100 ps. Such fast relaxation indicates that, in contrast to **TB2**, long-living triplets are not generated in the monomer as seen by the negligible signal in the ps-TAS upon 100 ps, further supporting the role of the DA unit in the **TBn** compounds to induce ISC. This further proves that the introduction of the DA unit to the **DCVT2** largely increases the triplet yield from 0 to 7%.

#### Mechanism of Intersystem Crossing

From the previous fluorescence emission data, obtained with different excitation wavelengths and therefore populating different singlet excited states, we observe no changes in the emission spectra and intensities, agreeing with the Kasha rule, which states that photophysical events all depart from the lowest singlet excited state. Therefore, the population of the triplet manifold from the lowest excited singlet state (S_1_ → T_*n*_) is controlled by the ISC rate, which is mainly determined by the adiabatic energy difference and spin–orbit coupling (SOC) constant between initial and final states.

**Figure 6:**
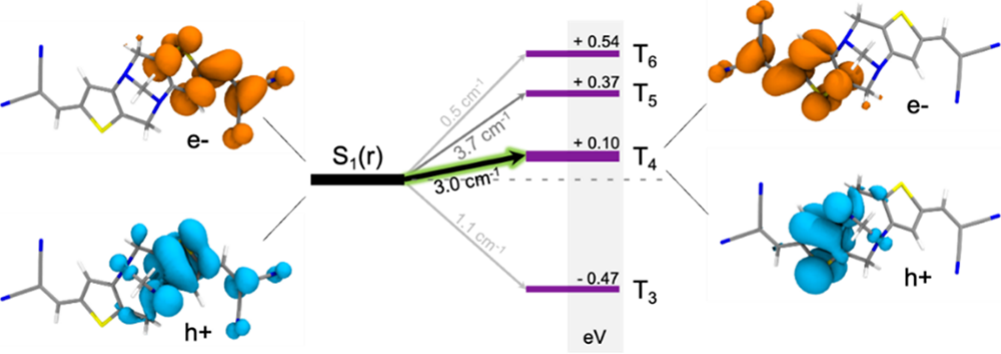
State energy (in eV) diagram of the TDDFT (CAM-B3LYP/6-311+G(d,p)) triplet states of **TB1** with respect to the relaxed S_1_ and associated S_1_ → T_*n*_ SOC values (cm^–1^). Electron/hole pair densities (orange/blue) are shown for the S_1_ and T_4_ states as obtained at the S_1_ equilibrium geometry.

Electronic structure calculations at the relaxed S_1_ geometry predict sizable SOC between S_1_ and the T_4_ and T_5_ states (≥3 cm^–1^) for **TB1** (Figure 6 and Table 1). Besides the high SOC constants, these two triplet states present the smallest vertical energy gaps with respect to S_1_, hence providing the necessary ingredients for efficient ISC. More specifically, these calculations suggest that ISC to the T_4_ triplet state is the most likely nonradiative transition given the rather small S_1_–T_4_ energy gap (0.10 eV) and significant value of the SOC constant (3 cm^–1^). Electron/hole pair densities for the S_1_ and T_4_ states of **TB1** (Figure 6) show that T_4_ is localized on the opposite side of S_1_ with yet sufficient overlap, in particular at the carbon atoms shared by the thiophene rings and the DA unit, to produce sizable SOC. Interestingly, whereas the hole density of both states (S_1_ and T_4_) in part delocalizes over the DA unit, the bridge does not contribute to the electron density of the transitions, which can be interpreted as an indication of some CT character in these states. It is also worth noticing the contribution of the orbitals of the DA nitrogen to the hole density of both states, suggesting their involvement in the ISC mechanism. This agrees with the absence of ISC in the monomer reference **DCVT2,** implying that the ISC effect relies on the central DA unit and its nitrogens. As the size of the system increases, the S_1_/T_4_ overlap vanishes (Figure S21) and the SOC values significantly drop (Table 1), which is in agreement with a decrease in the measured ISC rates. Moreover, a comparison of singlet–triplet energy gaps and SOCs in the monomer and dimer structures (Table S5) indicates the importance of the coupling of the two units in order to promote efficient ISC.

**Table 1:** TDDFT Energy Differences (Δ*E* in eV) between Pairs of Singlet and Triplet States of **TBn** Evaluated at the Relaxed S_1_ Geometry and Associated SOC constants

	**TB1**		**TB2**		**TB3**	
	Δ*E* (T_*n*_–S_1_)	SOC (cm^–1^)	Δ*E* (T_*n*_–S_1_)	SOC (cm^–1^)	Δ*E* (T_*n*_–S_1_)	SOC (cm^–1^)
T_1_	–1.74	2.9	–1.79	0.3	–1.66	0.2
T_2_	–1.18	0.5	–1.15	0.1	–0.93	0.0
T_3_	–0.47	1.1	–0.30	0.2	–0.53	0.1
T_4_	0.10	**3.0**	–0.01	**0.6**	–0.15	**0.1**
T_5_	0.37	3.7	0.49	0.9	0.47	0.3
T_6_	0.54	0.5	0.76	0.9	0.69	0.6
T_7_	0.76	0.1	0.77	1.8	0.92	0.9
T_8_	0.89	11.1	0.91	0.7	1.02	0.9

The selection rules for intersystem crossing, known as the El-Sayed rules,^37^ are based on the fact that spin transitions between states with the same orbital angular momentum are prohibited. On the contrary, transitions where the change in spin is compensated by a change in orbital angular momentum can trigger effective SOCs and fast ISCs. In practical terms, this latter statement implies that, to promote ISC, initial and final electronic states should involve orbital excitations of different character. This requirement has been demonstrated in countless occasions, such as in molecules exhibiting delayed fluorescence, in which ISC is activated by mixed excitations with local and charge transfer characters.^37−39^

**Figure 7:**
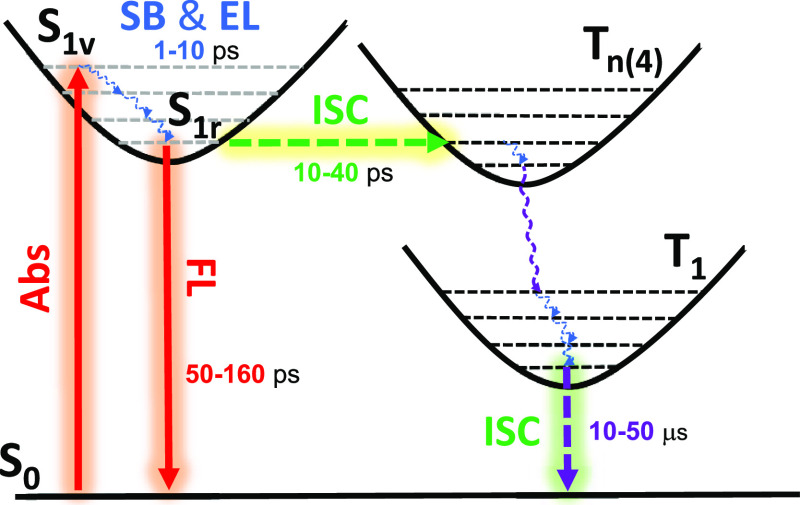
Jablonski diagram representing the photoinduced processes (**SB**: symmetry breaking, **EL**: electron localization, **FL**: fluorescence, **Abs**: absorption, **ISC**: intersystem crossing) in the **TBn** compounds. Decay lifetimes of the triplet excited states are for **TB2** and **TB3**.

In our Tröger’s base molecules, we have determined the partial CT character of the lowest excited singlet state and the symmetry-breaking exciton localization upon relaxation on the PES. The mixing of CT contributions in S_1_ is promoted by the dicyano groups, which act as stabilizers of the partial negative charge in the excited state, while the positive charge is stabilized by the joint role of the lone electron pairs of the nitrogen atoms and the methylene groups connected to the α-positions of the thiophene. Additionally, the excited state perpendicular disposition imparted by Tröger’s base enables direct TSI between the π-clouds of the two thiophenes that also contributes to stabilizing CT terms. According to our description, triplet states energetically close to S_1_, in particular the T_4_ state, are obtained through transitions between orbitals more localized on the thiophenes and dicyanovinylene moieties as the system size increases, that is, gradually gaining local excited state character. Hence, these triplet states can be termed as ^3^LE, but with a different spatial localization than in the relaxed S_1_. The nonzero singlet–triplet wave function overlap, combined with the presence of sulfur atoms, results in a rather strong SOC, triggering efficient singlet-to-triplet ISC in **TB1**, **TB2**, and **TB3**. The photophysical behavior interpreted from the experimental spectroscopic data together with calculations is represented in the Jablonski diagram in Figure 7.

## Conclusions

Three triads of acceptor-substituted oligothiophenes connected in almost-perpendicular disposition with Tröger’s base DA unit have been prepared and fully characterized by stationary and time-resolved electronic absorption and emission spectroscopies in conjunction with electronic structure calculations. We have shown that the Franck–Condon lowest energy lying excited states represent a delocalized transition with partial charge transfer character, where Tröger’s nitrogen atoms play a key role as electron donors. Symmetry-breaking modes activated along exciton relaxation localize the S_1_ state on one side of the molecule. The joint effect of TBI and TSI produces the fine alignment of the singlet and triplet excited states that are coupled, allowing for a very high intersystem crossing rate, which is further fueled by the different delocalized nature of the involved excited states. The enlargement of the oligothiophene bridge in **TB2** and **TB3** additionally modulates the singlet–triplet gaps, slowing the intersystem crossing rate, though still remarkable. The progressive separation of the donor–acceptor in the longer compounds triggers an overlap decrease and justifies the smaller SOCs in larger systems. Tröger’s base moiety is here presented as an unexploited building block to design new organic molecules and materials for applications in organic photonics.

The more relevant aspect of the photophysics of these triads is the efficient and ultrafast production of triplets, which is the result of a fine balance between through-bond and through-space couplings. In this investigation of the **TBn** triads, the overall mechanism revealed upon photoexcitation starts with an initial symmetry-breaking process in the S_1_ excited state, which consists in π-electron localization on the DCVTn units involving a charge transfer effect from the electro-releasing N atoms toward the acceptor DCV moieties. Both CT and local characteristics of the excitation promote the conditions for efficient intersystem crossing and fast population of triplets in **TBn**. The connection of excited state symmetry breaking and intersystem crossing by a localization and CT mechanism contributes to the understanding of the key electronic interactions in the excited manifold, which becomes of fundamental importance to benefit practical applications of organic chromophores in photonics such as in photovoltaic and in energy up-conversion. This dual effort in understanding the way of tuning electronic properties by going beyond those dictated by local through-bond connectivities, such as those emerging from through-space couplings, is remarkable and enlightens the necessity to holistically exploit molecular and functional features at all levels, in particular in excited states.

## Supporting Information Available

Full characterization including NMR spectra, IR spectra, and MALDI-HRMS data, DFT calculations, and transient absorption spectra and analysis (PDF)
