# Supplementary material for: V-Shaped Tröger Oligothiophenes Boost Triplet Formation by CT Mediation and Symmetry Breaking
Source: J Am Chem Soc. 2023 Dec 7;145(50):27295–306. doi: 10.1021/jacs.3c06916 (PMC10839832; doi:10.1021/jacs.3c06916)
Supplement: Supplementary file 1 — ja3c06916_si_001.pdf [file ja3c06916_si_001.pdf]

# Supporting Information

# V-Shaped Tröger Oligothiophenes Boost Triplets Formation by CT mediation and Symmetry Breaking

Samara Medina Rivero,[a,b,o] Matías J. Alonso-Navarro,[c,d,o] Claire Tonnelé,*[e,j,o] Jose M. Marín-Beloqui,[a] Fátima Suárez-Blas,[c,d] Tracey M. Clarke,[f] Seongsoo Kang,[g] Juwon Oh,[h] M. Mar Ramos,[d] Dongho Kim,*[g,i] David Casanova,*[e,j] José L. Segura*[c] and Juan Casado*[a]

[a]Department of Physical Chemistry, Faculty of Science, University of Málaga, 29071 Málaga, Spain

[b]Department of Physics & Astronomy, University of Sheffield, Sheffield S3 7RH, United Kingdom

[c]Organic Chemistry Department, Faculty of Chemistry, Complutense University of Madrid, 28040 Madrid, Spain

[d]Chemical and Environmental Technology Department, Rey Juan Carlos University, 28933, Madrid, Spain

[e]Donostia International Physics Center (DIPC), 20018 Donostia, Euskadi, Spain

[f]Department of Chemistry, University College London, London WC1H 0AJ, United Kingdom

[g]Department of Chemistry, Yonsei University, Seoul 03722, Korea

[h]Department of Chemistry, Soonchunhyang University, Asan 31538, Korea

[i]Division of Energy Materials, Pohang University of Science and Technology (POSTECH), Pohang 37673, Korea

[j]Ikerbasque Foundation for Science, 48009 Bilbao, Euskadi, Spain

[O] Equal contributions as first authors.

## Table of Contents

1. Synthesis and characterization ........................................................S2
2. Quantum chemical calculations *cis* conformer ...................................S12
3. Transient electronic absorption spectroscopy ......................................S17
4. Quantum chemical calculations for the *trans* conformers ....................S19
5. References ..........................................................................................S21

# 1. Synthesis and Characterization

All the chemicals were purchased from commercial suppliers and used without further purification. Compounds **3**,[1] **4**,[2] **TB**[3] and **TB-CHO**[3] were obtained as previously described or with some modifications. The thiophene-based analogue **DCVT2** was synthetized according to the literature.[2] $^1$H-NMR and $^{13}$C-NMR spectra were recorded on a Bruker Avance 300 MHz spectrometer. Chemical shifts are reported in ppm and referenced to the residual non-deuterated solvent frequencies ($CDCl_3$: δ 7.26 ppm for $^1$H, δ 77.0 ppm for $^{13}$C, Acetone-*d6*: δ 2.05 ppm for $^1$H, δ 29.8 and 206 ppm for $^{13}$C, DMSO-*d6*: δ 2.50 ppm for $^1$H, and 39.5 ppm for $^{13}$C).

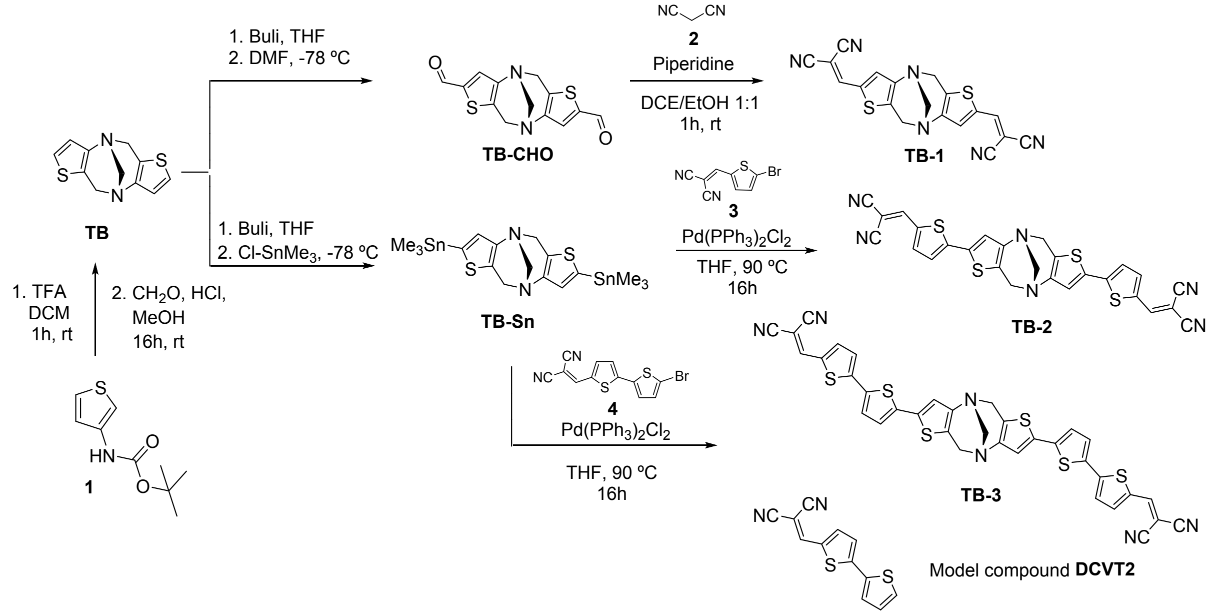


**Scheme S1.** Synthetic route described in the article for the Trögger base analogues **TB1**, **TB2** and **TB3** and the model compound **DCVT2.**

### (4R,9R)-5,10-dihydro-4,9-methanodithieno[3,2-b:3',2'-f][1,5]diazocine – TB

Under Ar atmosphere, **1** (1g, 5,4 mmol) is suspended in 10 mL of dry dichloromethane. The mixture is cooled to 0 ºC and 7,5 mL of TFA is added, as it is described previously.[4] The reaction is stirred 1h at room temperature and then the solvent is removed under reduced pressure. The obtained crude is dissolved in 6 mL of MeOH and 2,22 mL of HCl (37%) and 2,53 mL of $CH_2O$ are added to the reaction. The mixture is stirred at 27 ºC overnight and once the time is complete the crude is poured into aqueous ammonia solution (4%) and extracted with dichloromethane. The combined organic phases were washed with brine and dried over $MgSO_4$. After removal the solvent, the crude was washed several times with cold methanol to obtain the pure product **TB** as a white solid (31%).

$^1$H-NMR (300 MHz, $CDCl_3$): δ (ppm) = 7.11 (d, 5.3 Hz, 1H), 6.83 (d, J = 5.3 Hz, 1H), 4.58 (d, J = 16 Hz, 1H), 4.23 (s, 1H), 4.13 (d, J = 16 Hz, 1H).

$^{13}$C-NMR (75 MHz, $CDCl_3$): δ (ppm) = 144.86, 123.24, 123.10, 122.98, 68.00, 53.68.

### (4R,9R)-2,7-bis(trimethylstannyl)-5,10-dihydro-4,9-methanodithieno[3,2-b:3',2'-f][1,5]diazocine – TB-Sn

To a solution of **TB** (100 mg, 0.43 mmol) in 6 mL of dry THF at 0 ºC a solution of n-BuLi (0.8 mL, 1.6 M in hexane) was added and the mixture was stirred 30 min. at room temperature and then cooled to -78 ºC. To the cooled solution $ClSnMe_3$ (255mg, 1.5 eq., in 2 mL of dry THF) was added and the solution was warmed up to room temperature slowly and stirred for 30 min. at room temperature. Then, water was added to the reaction, extracted with dichloromethane (x3) and the

organic phases were washed with brine and dried over $MgSO_4$. After removal of the solvent, the organic oily residue was used without further purification.

$^1$H-NMR (300 MHz, $CDCl_3$): δ (ppm) = 6.89 (s, 1H), 4.59 (d, J = 16 Hz, 1H), 4.19 (d, J = 16 Hz, 1H), 4.15 (s, 1H), 0.32 (s, 9H).

**(4R,9R)-5,10-dihydro-4,9-methanodithieno[3,2-b:3',2'-f][1,5]diazocine-2,7-dicarbaldehyde – TB-CHO**

To solution of **TB** (100 mg, 0.43 mmol) in 6 mL of dry THF at 0 ºC a solution of n-BuLi (0.8 mL, 1.6 M in hexane) was added and the mixture was stirred 30 min. at room temperature and then cooled to -78 ºC. To the cooled solution N,N-dimethylformamide (255 mg, 1.5 eq., in 2 mL of dry THF) was added and the solution was warmed up to room temperature slowly and stirred for 30 min. at room temperature. Then, water was added to the reaction, extracted with dichloromethane (x3) and the organic phases were washed with brine and dried over $MgSO_4$. After removal of the solvent, the residue was purified by chromatography column in Hexane/Ethyl Acetate 1:2, to give the product **TB-CHO** as a yellowish solid (24mg, 18%).

$^1$H-NMR (300 MHz, $CDCl_3$): δ (ppm) = 9.82 (s, 1H), 7.52 (s, 1H), 4.66 (d, J = 16 Hz, 1H), 4.28 (s, 1H), 4.23 (d, J = 16 Hz, 1H).

$^{13}$C-NMR (75 MHz, $CDCl_3$): δ (ppm) = 182.3, 146.5, 140.7, 134.3, 131, 67.6, 54.4.

**2,2'-(((4R,9R)-5,10-dihydro-4,9-methanodithieno[3,2-b:3',2'-f][1,5]diazocine-2,7-diyl)bis(methanylylidene))dimalononitrile – TB1**

To solution of **TB-CHO** (100 mg, 0.43 mmol) in a mixture of DCE/EtOH 1:1 at room temperature it was added 98 mg of **2** and 1 drop of piperidine and the reaction was stirred 30min. once the time is over, the reaction is washed with deionized water and extracted with chloroform. The organic phases were washed with brine and dried over $MgSO_4$. The obtained solid was purified by column chromatography (Silica gel flash, chloroform/methanol 90:1), obtaining 34 mg of **TB1** (53mg, 40%) as a yellow powder.

$^1$H-NMR (300 MHz, Acetone-*d*6): δ (ppm) = 8.32 (d, J = 0.6 Hz, 1H), 7.77 (d, J = 0.6 Hz, 1H), 4.80 (d, J = 18.0 Hz, 1H), 4.48 (d, J = 18.0, 1H), 4.37 (m, 1H).

$^{13}$C-NMR (75 MHz, Acetone-*d*6): δ (ppm) = 152.71, 148.34, 137.98, 135.86, 133.53, 114.94, 114.21, 79.20, 77.24, 67.83, 55.20.

FTIR (ATR, $CHCl_3$): υ ($cm^{-1}$): 2990, 2917, 2874, 2211, 1694, 1432, , 1235.

MALDI-HRMS (m/z): calculated for $C_{19}H_{10}N_6S_2$: 386.0408, found ($M^+$): 386.0422.

**2,2'-((5,5'-((4R,9R)-5,10-dihydro-4,9-methanodithieno[3,2-b:3',2'-f][1,5]diazocine-2,7-diyl)bis(thiophene-5,2-iyl))bis(methanylylidene)) dimalononitrile – TB2**

A solution of **TB-Sn** (65 mg, 0.011 mmol), **3** (61 mg, 0.025 mmol, 2.2 eq.) and $Pd(Ph_3)_2Cl_2$ (0.9 mg, 0.0011 mmol, 0.1 eq) in 10 mL of dry THF is stirred overnight at 85 ºC. The reaction is cooled, the solvent volume is half-reduced and then 20 mL of hexane is added. The emerging solid was filtered and washed several times with hexane and methanol to obtain the pure product **TB2** as an orange solid (44 mg, 65%).

$^1$H-NMR (300 MHz, Acetone-*d*6): δ (ppm) = 8.37 (s, 1H), 7.88 (d, J = 4.1 Hz, 1H), 7.46 (s, 1H), 7.45 (d, J = 4.1 Hz, 1H), 4.64 (d, J = 17.1 Hz, 1H), 4.38 (d, J = 17.2 Hz, 1H), 4.30 (s, 1H).

$^{13}$C-NMR (75 MHz, DMSO-*d*6): δ (ppm) = 152.40, 148.07, 146.91, 142.49, 133.27, 131.20, 127.00, 124.97, 123.90, 114.68, 113.96, 74.21, 53.12.

FTIR (ATR, $CHCl_3$): υ ($cm^{-1}$): 2995, 2916, 2870, 2212, 1694, 1615, 1435, 1322, 1235.

MALDI-HRMS (m/z): calculated for $C_{27}H_{14}N_6S_4$: 550.0163, found ($M^+$): 550.0170.

**2,2'-((5',5'''-((4R,9R)-5,10-dihydro-4,9-methanodithieno[3,2-b:3',2'-f][1,5]diazocine-2,7-diyl)bis([2,2'-bithiophene]-5',5-diyl))bis(methanylylidene)) dimalononitrile – TB3**

A solution of **TB-Sn** (100 mg, 0.18 mmol), **4** (126 mg, 0.39 mmol, 2.2 eq.) and $Pd(Ph_3)_2Cl_2$ (12 mg, 0,02 mmol, 0.1 eq) in 10 mL of dry THF is stirred overnight at 85 ºC. The reaction is cooled, the solvent volume is half-reduced and then 20 mL of hexane is added. The emerging solid is filtered and washed several times with hexane and methanol to obtain the pure product **TB3** as an orange solid (66 mg, 51%).

$^1$H-NMR (300 MHz, Acetone-*d*6): δ (ppm) = 8.39 (s, 1H), 7.92 (d, J = 4.0 Hz, 1H), 7.58 (d, J = 4.0 Hz, 1H), 7.56 (d, J = 4.1 Hz, 1H), 7.27 (d, J = 4.0 Hz, 1H), 7.23 (s, 1H), 4.60 (d, J = 17.0 Hz, 1H), 4.34 – 4.25 (m, 2H).

$^{13}$C-NMR (75 MHz, Acetone-*d*6): δ (ppm) = 152.21, 148.69, 142.61, 141.03, 134.71, 134.36, 129.33, 125.77, 125.23, 124.14, 123.64, 122.22, 115.19, 114.57, 79.20, 76.28, 68.40, 54.01.

FTIR (ATR, $CHCl_3$): υ ($cm^{-1}$): 2995, 2917, 2875, 2210, 1694, 1615, 1432, 1375, 1312, 1235, 1175.

MALDI-HRMS (m/z): calculated for $C_{35}H_{18}N_6S_6$: 713.9917, found ($M^+$+1): 714.9997

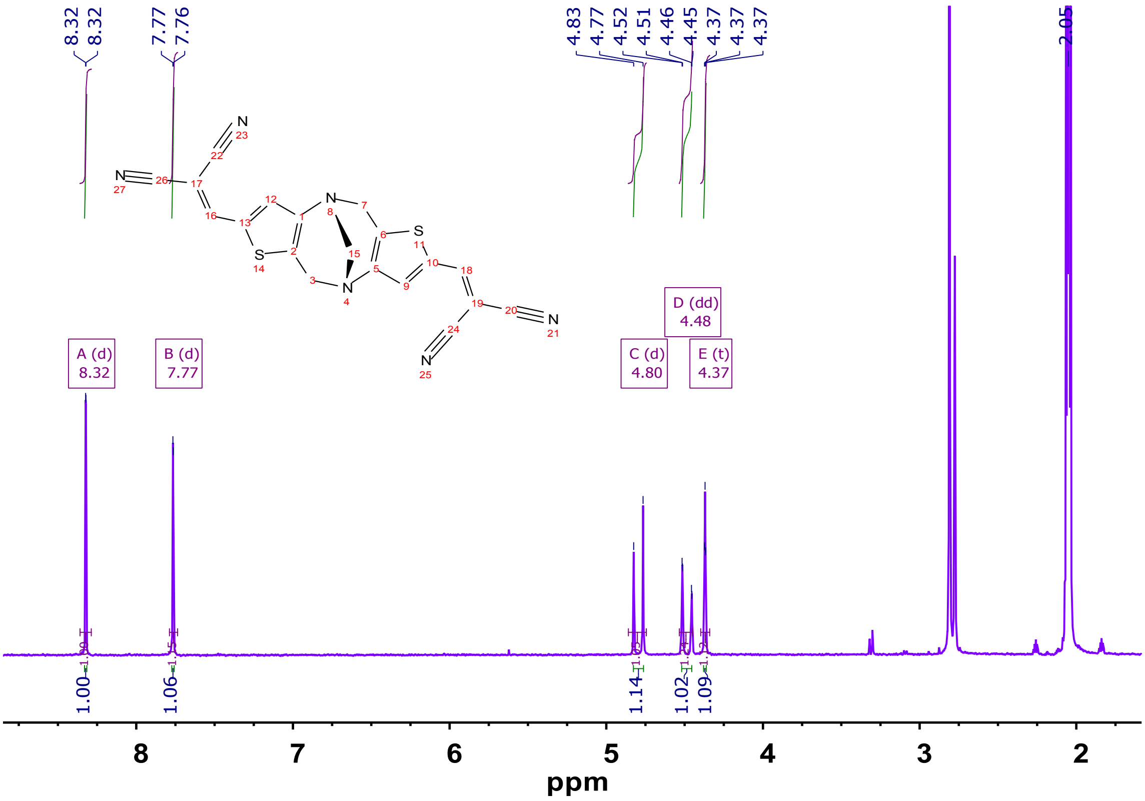

**Figure S1.** $^1$H-NMR spectrum of **TB1** in Acetone-*d6*.

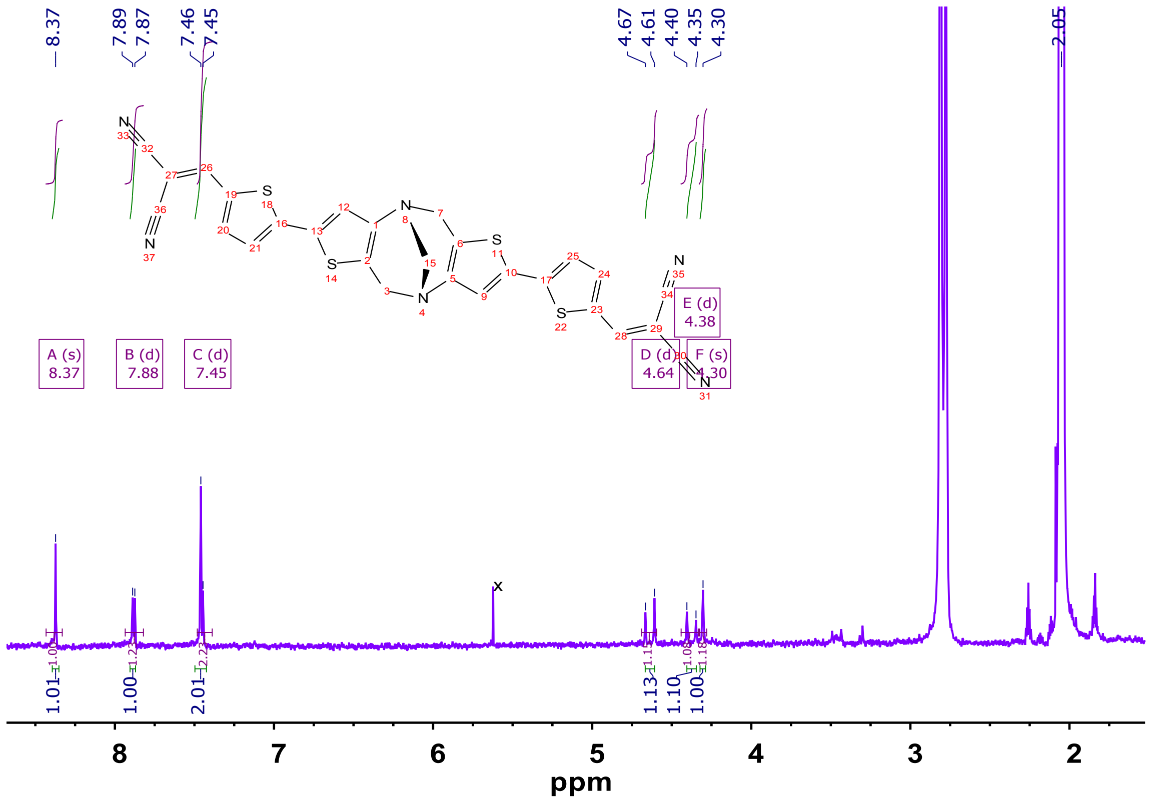


**Figure S2.** $^{1}$H-NMR spectrum of **TB2** in Acetone-*d6*.

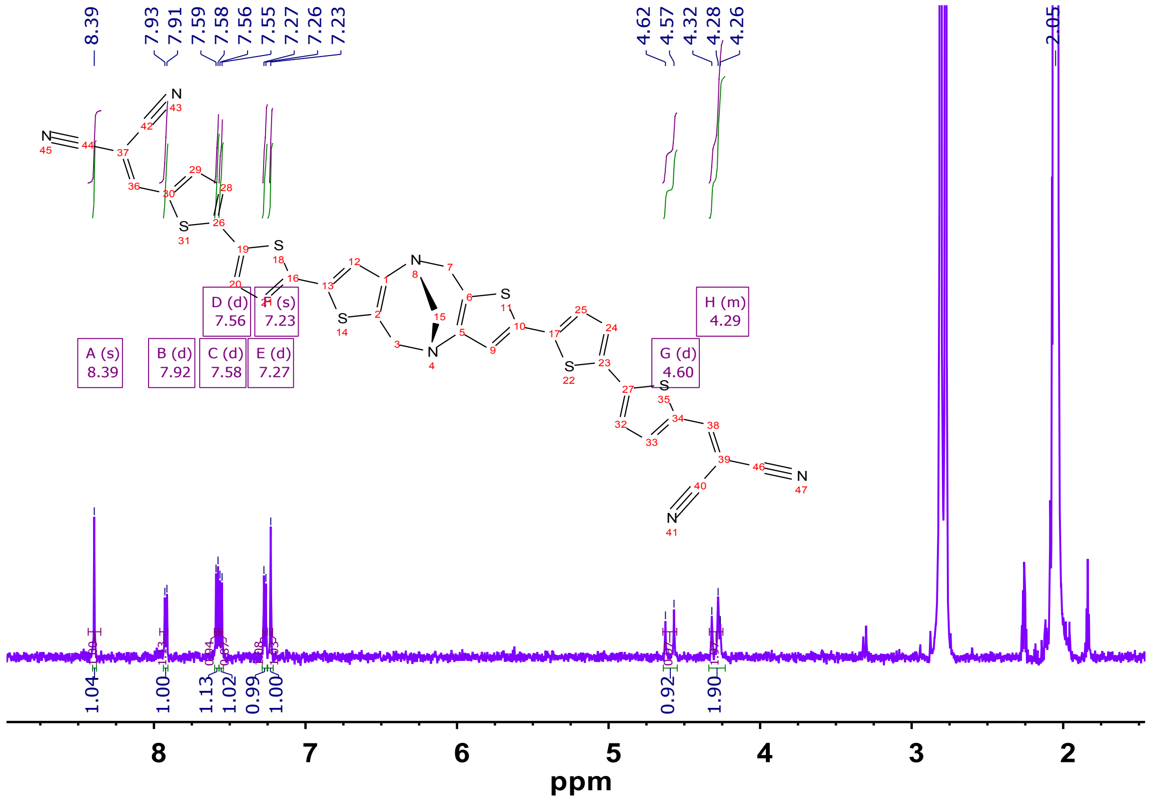


**Figure S3.** $^{1}$H-NMR spectrum of **TB3** in Acetone-*d6*.

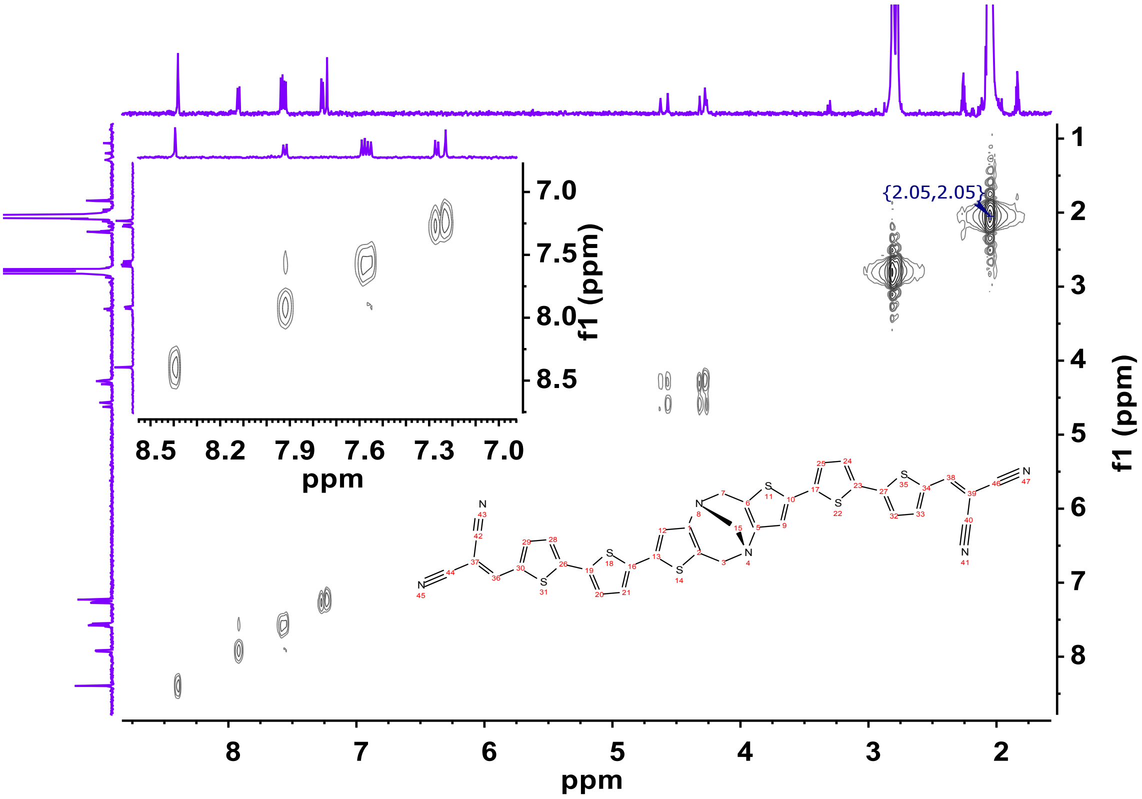


**Figure S4.** COSY $^1H$-$^1H$-NMR spectrum of **TB3** in Acetone-*d6*.

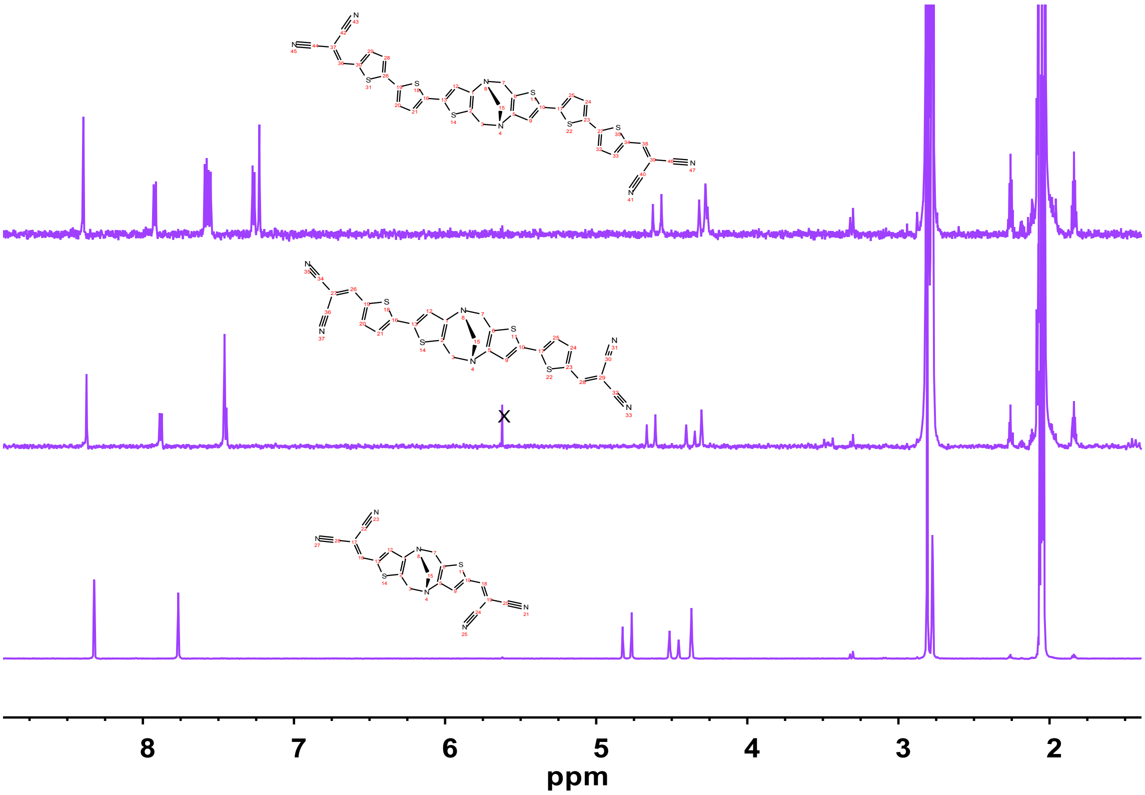


**Figure S5.** $^1H$-NMR spectrum comparison of **TB1**, **TB2** and **TB3** in Acetone-*d6*.

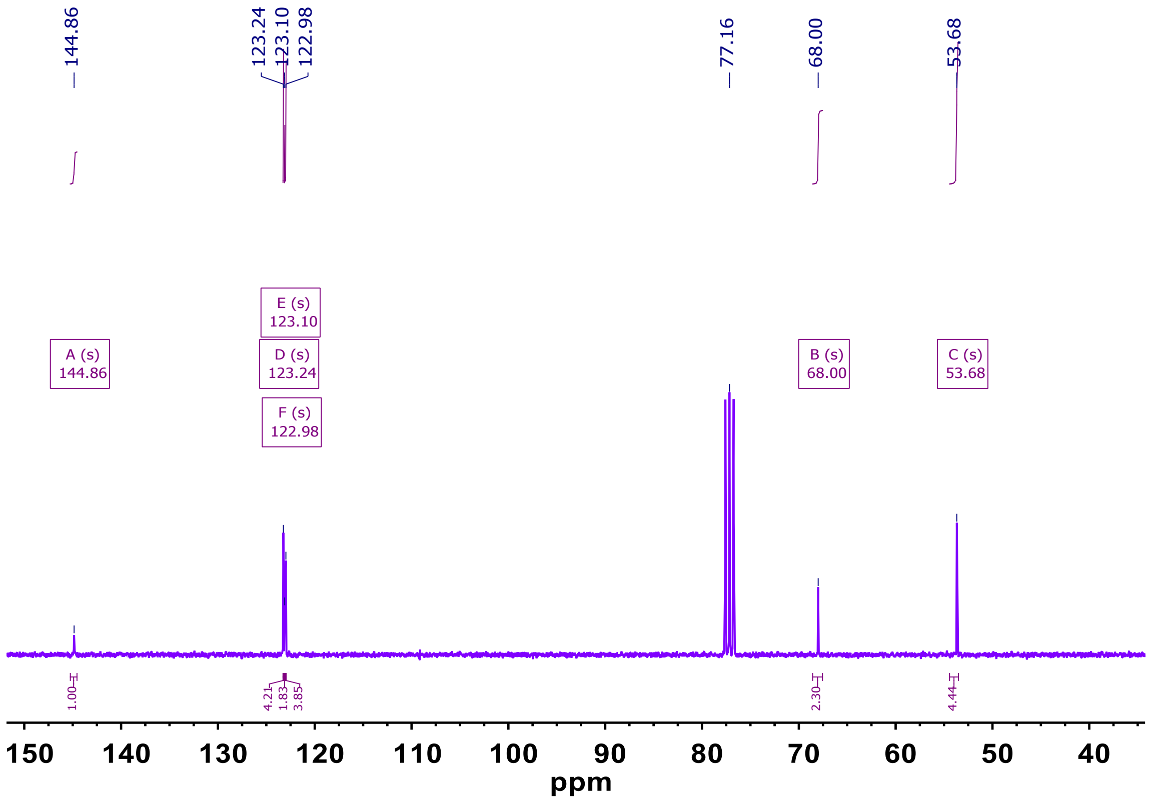

**Figure S6.** $^{13}$C-NMR spectrum of **TB** in $CDCl_3$.

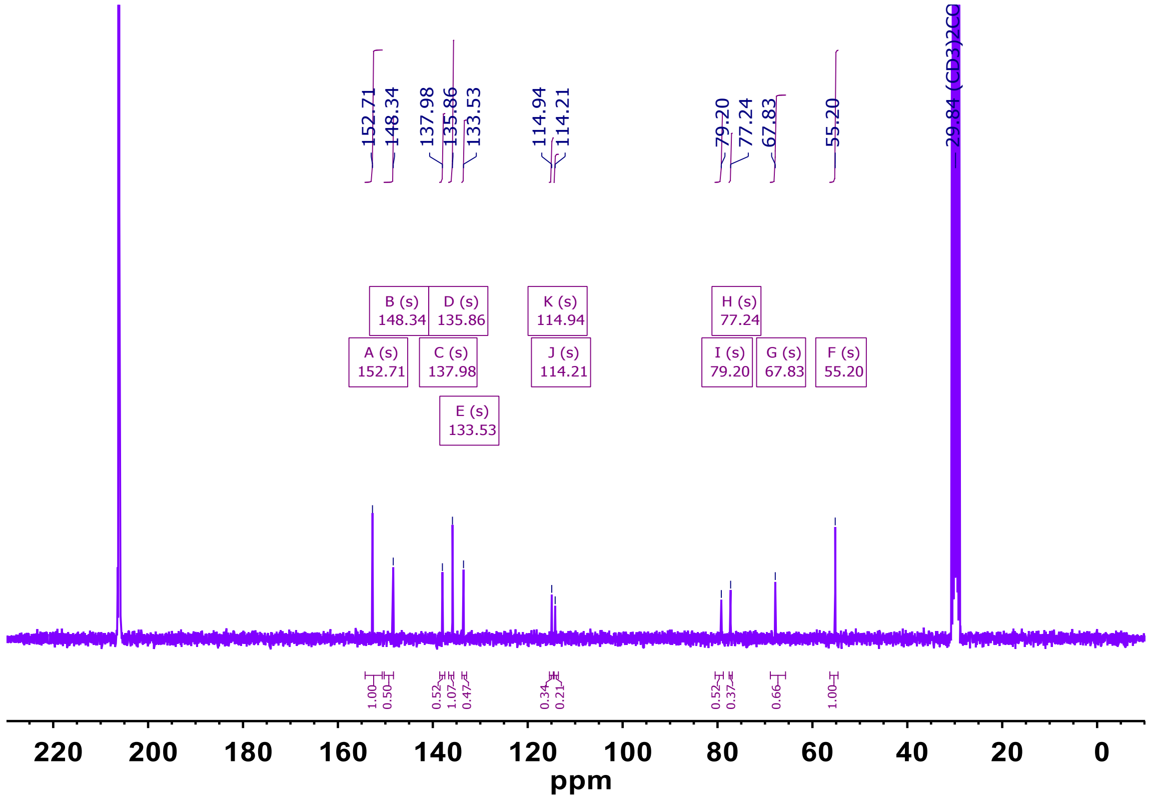

**Figure S7.** $^{13}$C-NMR spectrum of **TB1** in Acetone-*d6*.

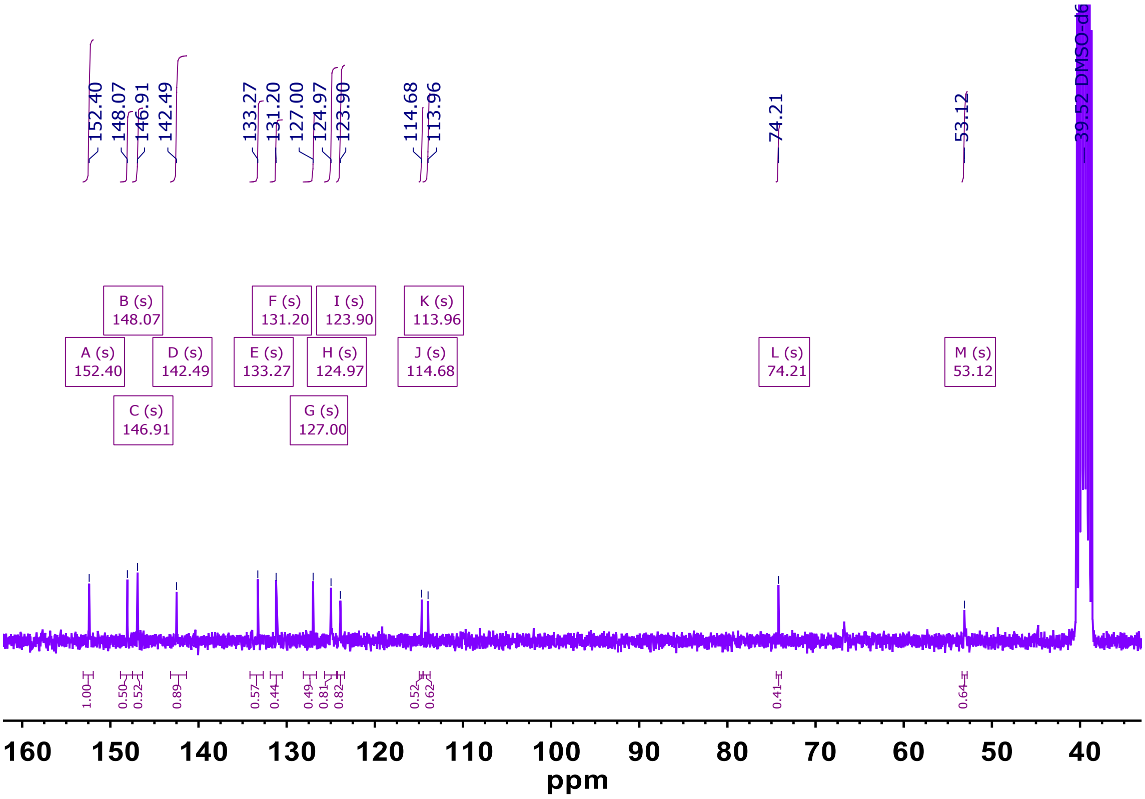


**Figure S8.** $^{13}$C-NMR spectrum of **TB2** in DMSO-*d6*.

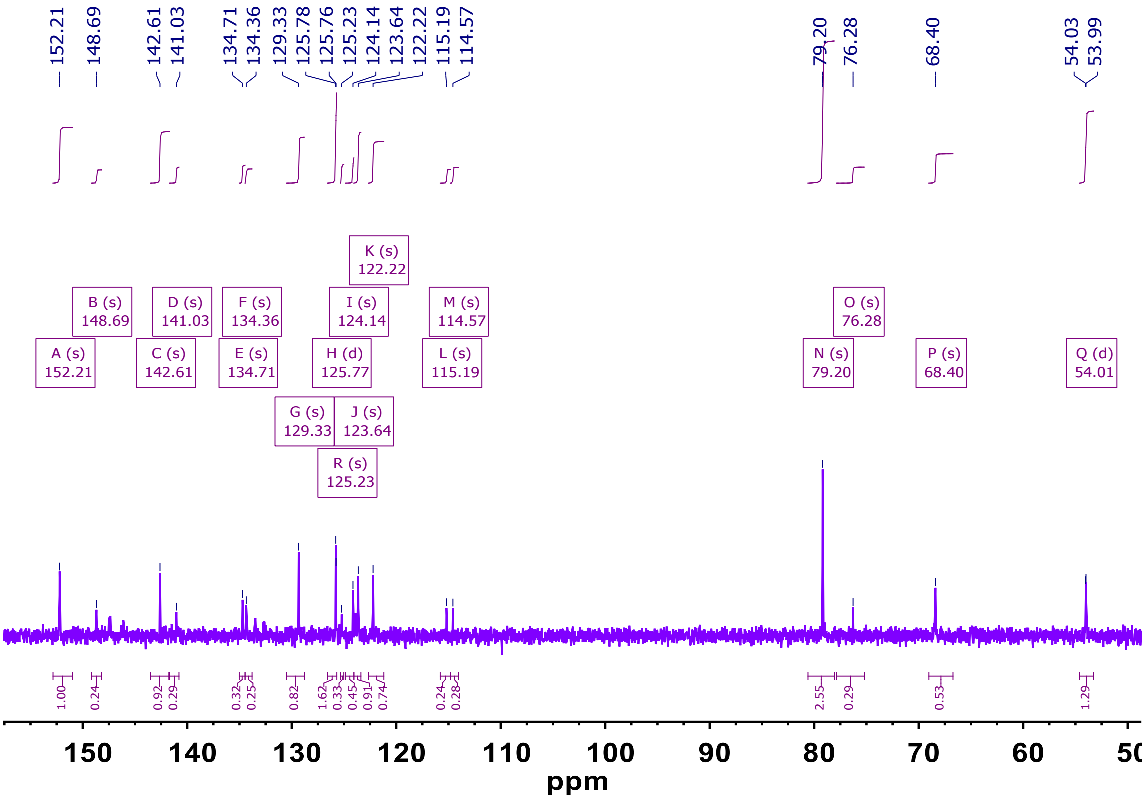


**Figure S9.** $^{13}$C-NMR spectrum of **TB3** in Acetone-*d6*.

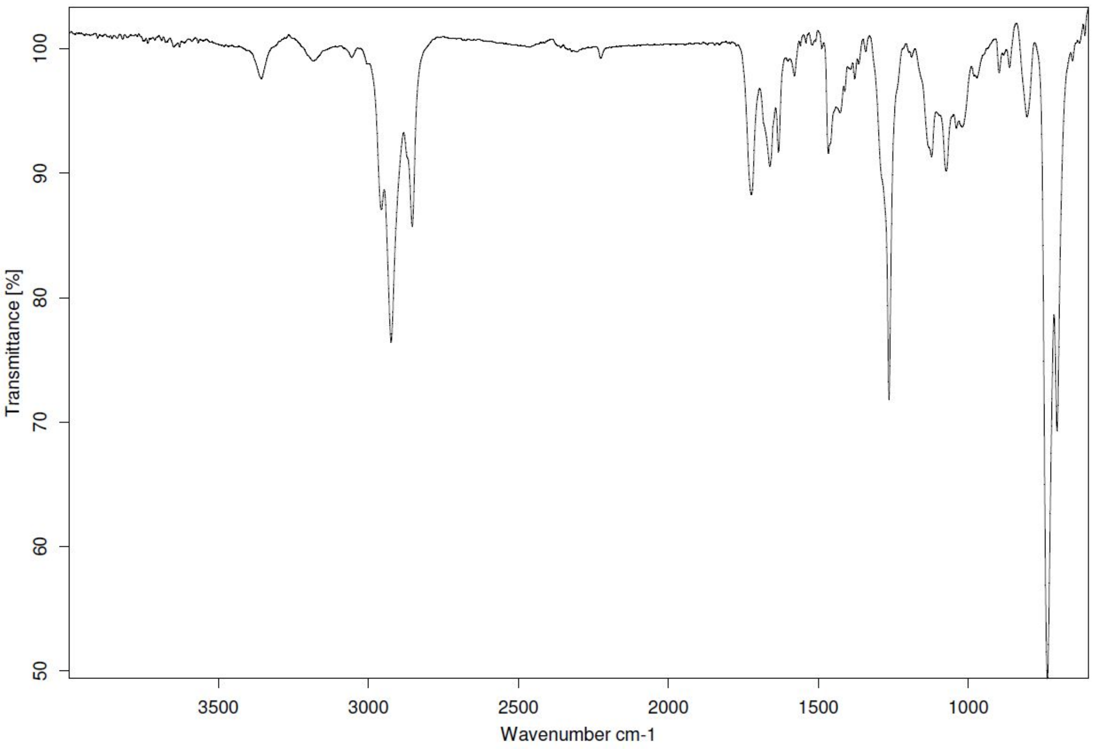


**Figure S10.** IR spectrum of **TB1**.

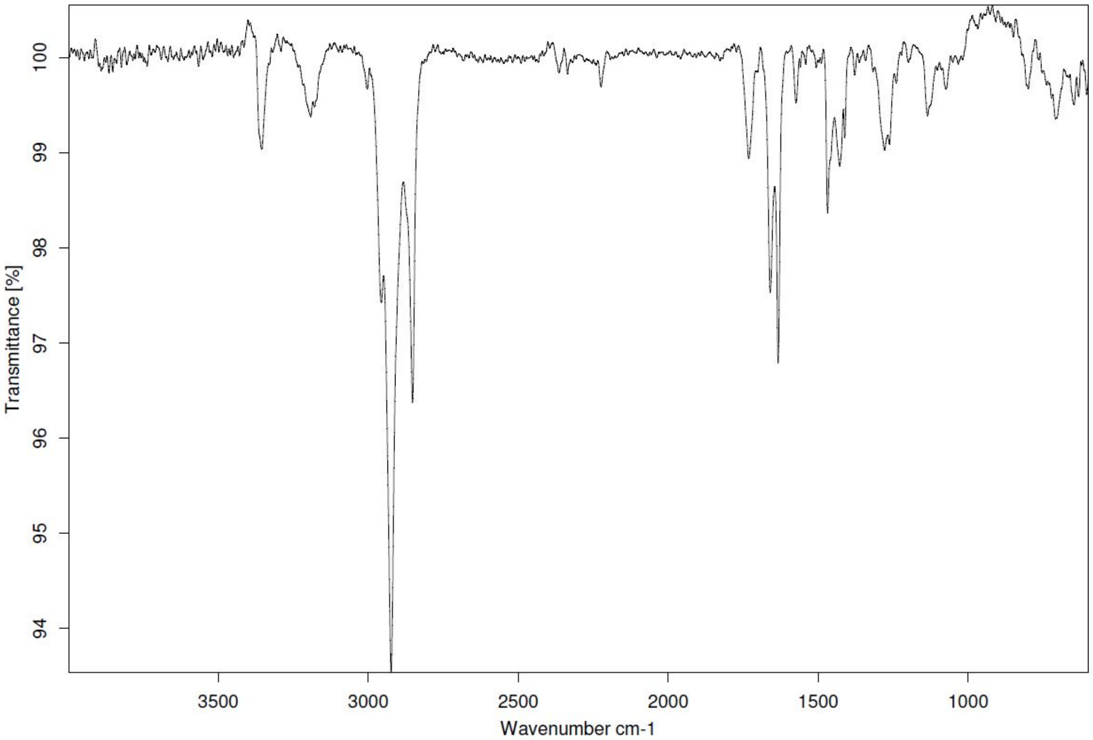


**Figure S11.** IR spectrum of **TB2**.

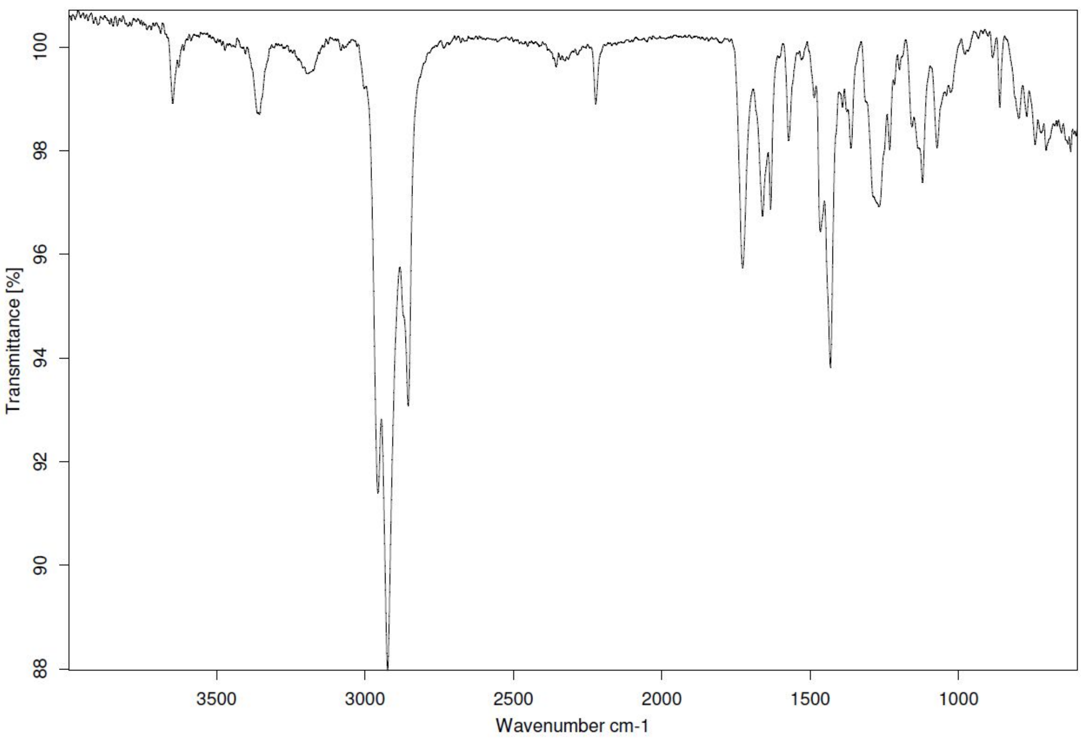


**Figure S12.** IR spectrum of **TB3**.

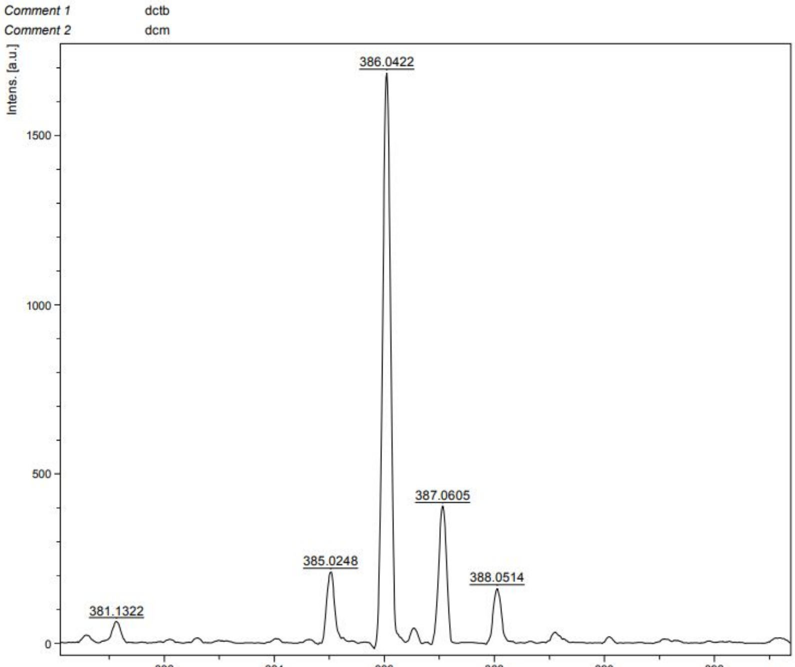


**Figure S13.** MALDI-HRMS (m/z) spectrum of **TB1**.

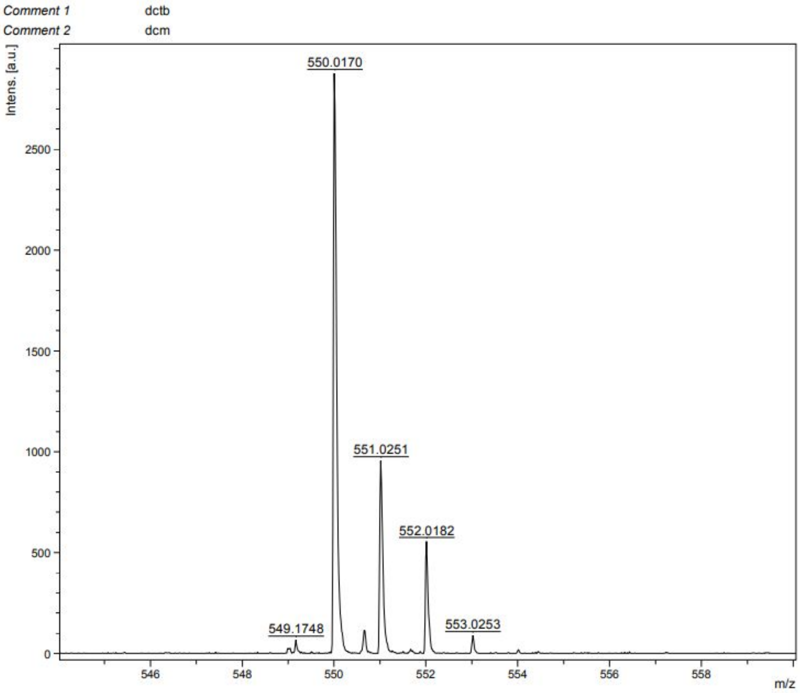


**Figure S14.** MALDI-HRMS (m/z) spectrum of **TB2**.

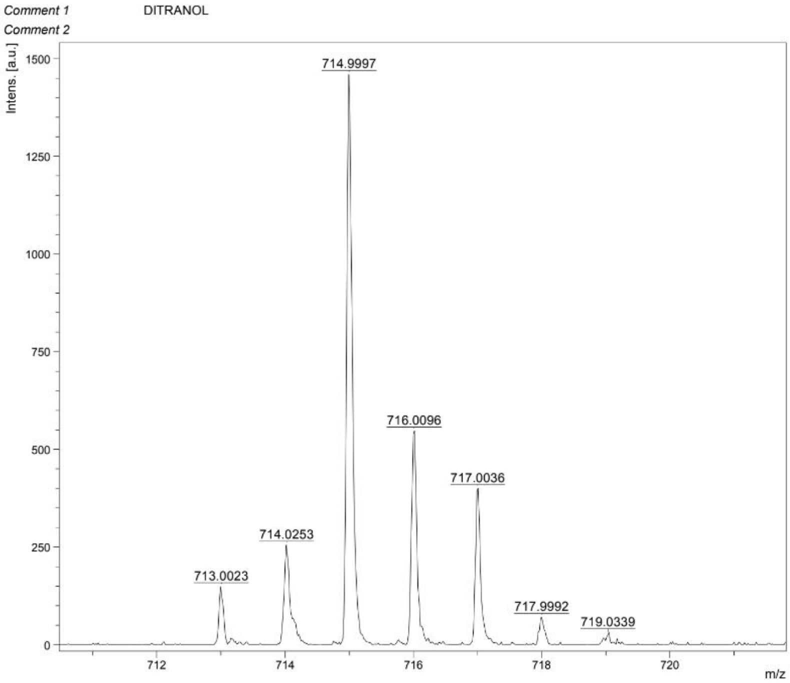


**Figure S15.** MALDI-HRMS (m/z) spectrum of **TB3**.

# 2. Quantum Chemical Calculations

## Computational Details

Ground and excited states geometries were optimized within the framework of the density functional theory (DFT) at the CAM-B3LYP/6-31g(d,p) level. Excitation energies and interstate spin-orbit coupling were computed using time-dependent DFT (TDDFT) at the CAM-B3LYP /6-311+g(d,p) level, with and without resorting to the Tamm-Dancoff approximation (TDA). Electron-hole correlation plots for the characterization of excited states were obtained using TheoDORE 3.0.[5] All calculations were performed using Gaussian 16 and Q-Chem program packages.[6, 7]

## Structural characterization: ground state

Selected relevant geometrical parameters are defined in Figure S16 and reported for all compounds investigated in Table S1.

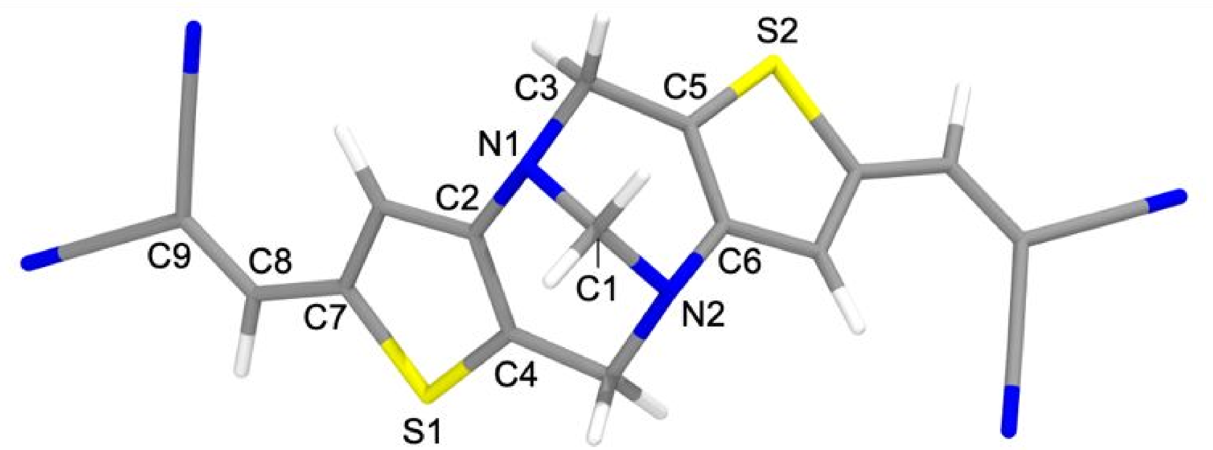


**Figure S16.** Definition of the geometrical parameters: $\theta_1$ = N1-C1-N2, $\theta_2$ = C2-N1-C3, $\theta_3$ = C4-C1-C5, $\theta_4$ is defined as the angle between vectors S1-C4 and C5-S2, the twist of DCVT unit is evaluated by the dihedral angle $\theta_5$ = C2-C4-C6-C5, the deviation from planarity within the DCVT unit is characterized by the dihedral angle $\theta_6$ = S1-C7-C8-C9.

**Table S1.** Structural parameters at the ground state geometry.

| | TB1 | TB1-2H$^+$ | TB2 | TB3 |
|---|---|---|---|---|
| $\theta_1$ | 111.5 | 107.2 | 111.7 | 111.8 |
| $\theta_2$ | 113.4 | 113.5 | 113.2 | 113.2 |
| $\theta_3$ | 81.0 | 78.1 | 81.0 | 81.1 |
| $\theta_4$ | 78.1 | 81.3 | 77.8 | 78.3 |
| $\theta_5$ | -24.5 | -26.7 | -24.8 | -24.6 |
| $\theta_6^a$ | -176.0 | -173.8 | -162.0 | -157.7 |

$^a\theta_6$ for **TB2** and **TB3** corresponds to the dihedral angle between the two first thiophene units of DCVT.

## Conformers

**Table S2.** Relative energies (in kcal.mol$^{-1}$) of the cis and trans conformers of **TB1**, **TB2** and **TB3**.

| (kcal.mol$^{-1}$) | TB1 | TB2 | TB3 |
|---|---|---|---|
| trans | 0.43 | 0.00 | 0.00 |
| cis | 0.00 | 1.24 | 0.80 |

## TDDFT data (ground state geometry)

**Table S3.** Vertical transition energies (in eV), oscillator strengths (in parentheses) and orbital contributions of monomers **DCVT1** and **DCVT2**, and molecules **TB1**, **TB2** and **TB3** calculated at the TDDFT level, and experimental values corresponding to the absorption maxima.

| | state | exp | TDDFT | contributions |
|---|---|---|---|---|
| **DCVT1** | $S_1$ | / | 3.99 (0.62) | 98% H→L |
| TB1 | $S_1$ | 3.12 | 3.60 (0.90) | 72% H→L + 25% H-1→L+1 |
| | $S_2$ | / | 3.73 (0.14) | 56% H→L+1 + 40% H-1→L |
| | $S_3$ | 3.83 | 4.09 (0.28) | 66% H-2→L + 15% H-3→L+1 + 10% H-1→L+1 + 6% H→L |
| | $S_4$ | / | 4.20 (0.10) | 52% H-2→L+1 + 20% H-1→L + 17% H-3→L + 9% H→L+1 |
| **DCVT2** | $S_1$ | / | 3.32 (0.95) | 96% H→L |
| TB2 | $S_1$ | 2.75 | 3.15 (1.65) | 54% H→L + 38% H-1→L+1 |
| | $S_2$ | / | 3.30 (0.48) | 49% H→L+1 + 44% H-1→L |
| TB3 | $S_1$ | 2.59 | 2.92 (2.08) | 47% H→L + 38% H-1→L+1 |
| | $S_2$ | / | 3.03 (0.72) | 44% H→L+1 + 42% H-1→L |

## Molecular orbitals at the ground state geometry

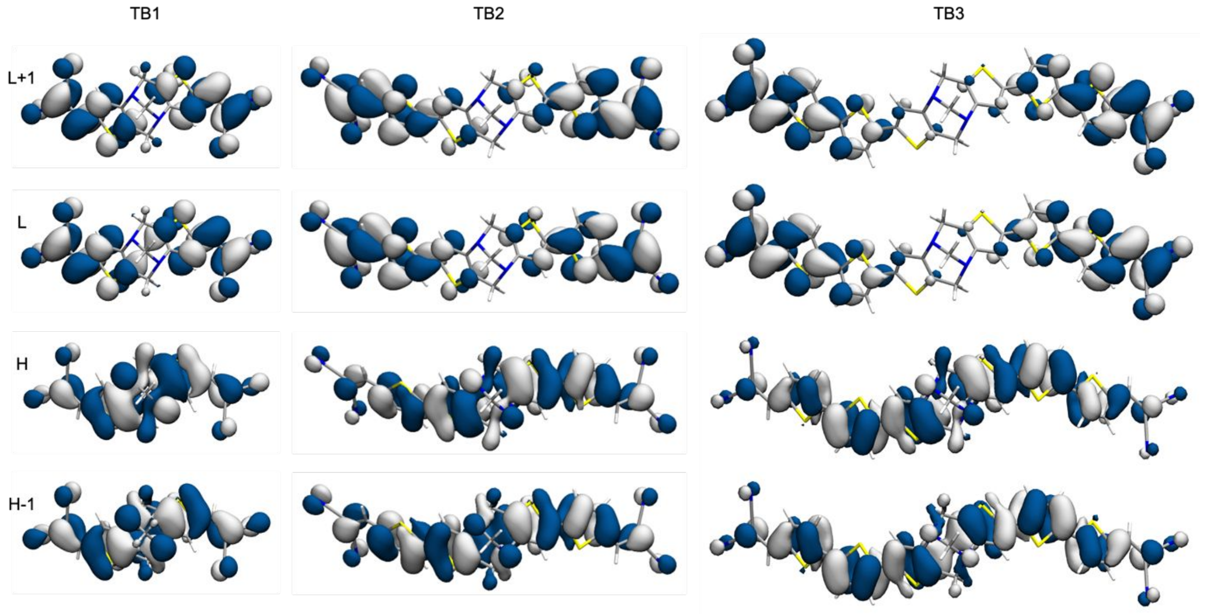


**Figure S17.** Molecular orbitals (HOMO-1, HOMO, LUMO and LUMO+1) involved in the excitations to the lowest singlet excited states of **TB1**, **TB2** and **TB3**.

## Point dipole approximation

Exciton coupling for oblique arrangement of the transition dipoles can be evaluated using the point-dipole approximation (PDA) as:

$$V_{12}^{PDA} = \frac{1}{4\pi\epsilon_0}\left[\frac{\boldsymbol{\mu}_1 \cdot \boldsymbol{\mu}_2}{r_{12}^3} - 3\frac{(\boldsymbol{\mu}_1 \cdot \boldsymbol{r}_{12})(\boldsymbol{\mu}_2 \cdot \boldsymbol{r}_{12})}{r_{12}^5}\right]$$

where $\mathbf{r}_{12}$ is the vector distance between molecules 1 and 2, and $\boldsymbol{\mu}_1$ and $\boldsymbol{\mu}_2$ their respective transition dipole moments.

The following parameters have been used for the calculation:

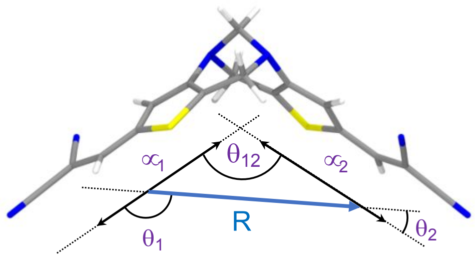


$\boldsymbol{\mu}_1$ = (-2.627  -1.429  -1.105)
$\boldsymbol{\mu}_2$ = (2.628  -1.427  -1.106)
$\mathbf{r}_{12}$ = (7.688  -0.350  -0.699)
$\theta_1$ = 140°
$\theta_2$ = 29°
$\theta_{12}$ = 111°

**Figure S18.** Dipole-dipole interaction in **TB1** and associated parameters.

The Davydson splitting, i.e., the energy gap between the two lowest excited singlet states of the dimer, is then obtained as twice the exciton coupling of $\boldsymbol{\mu}_1$ and $\boldsymbol{\mu}_2$ and amounts to 0.045 eV.

## e-h correlation plots (ground state geometry)

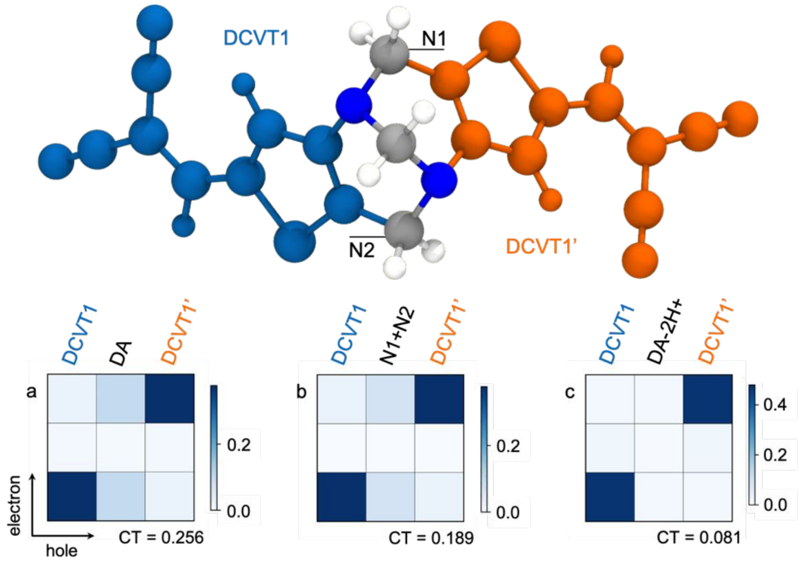


**Figure S19.** Electron-hole correlation plots for the $S_1$ state of (a) **TB1** considering DA as central fragment (without CC units of thiophene), (b) **TB1** considering only **TB**'s nitrogen atoms as central fragment, and of (c) the protonated **TB1**.

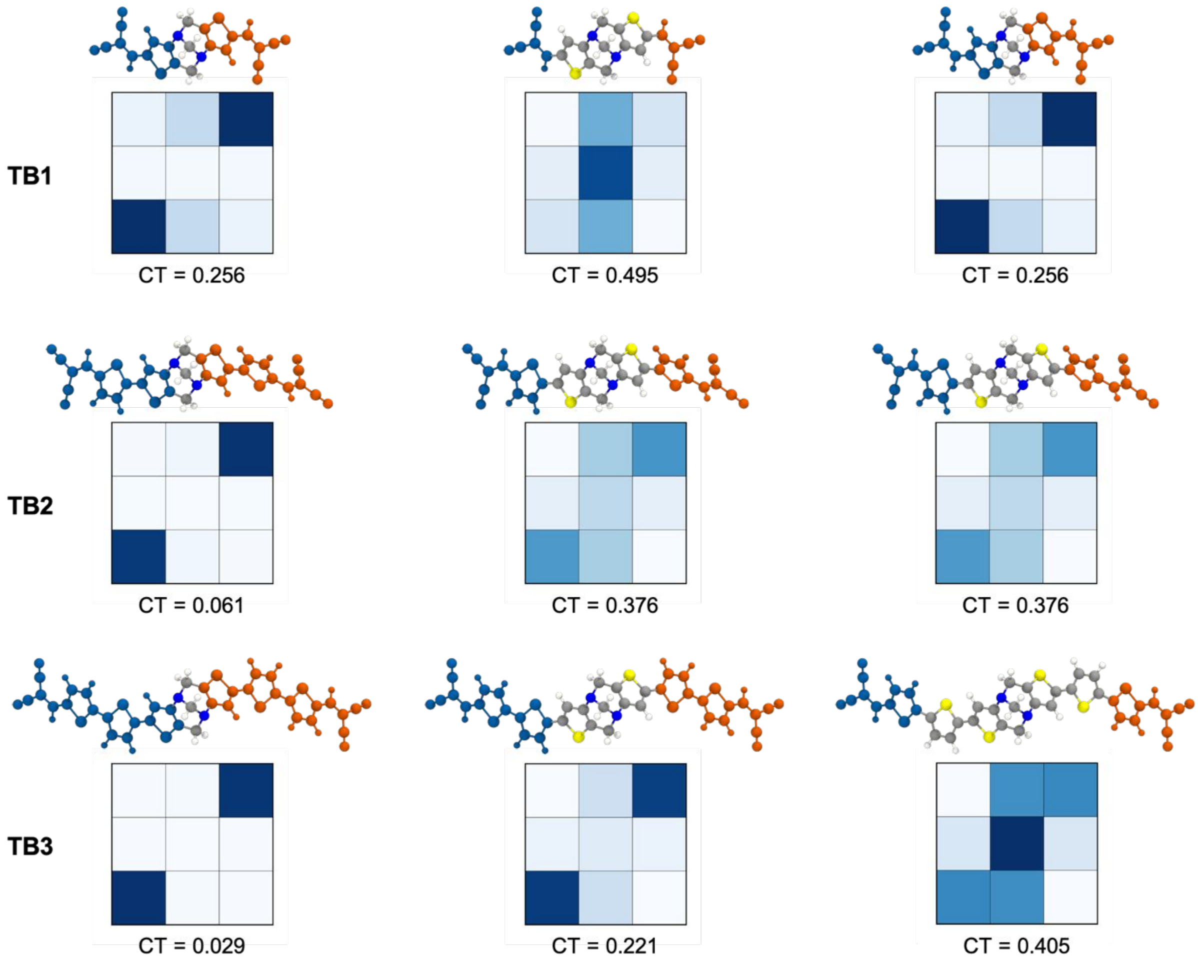


**Figure S20.** Electron-hole correlation plots for the $S_1$ state of **TB1**, **TB2** and **TB3** considering different fragmentation schemes: (left) defining the central fragment as the DA, without CC units of thiophene, (center) defining the central fragment as **TB** (DA + 2 thiophenes), (right) defining the side fragments as DCVT1.

## e-h pair densities ($S_1$ geometry)

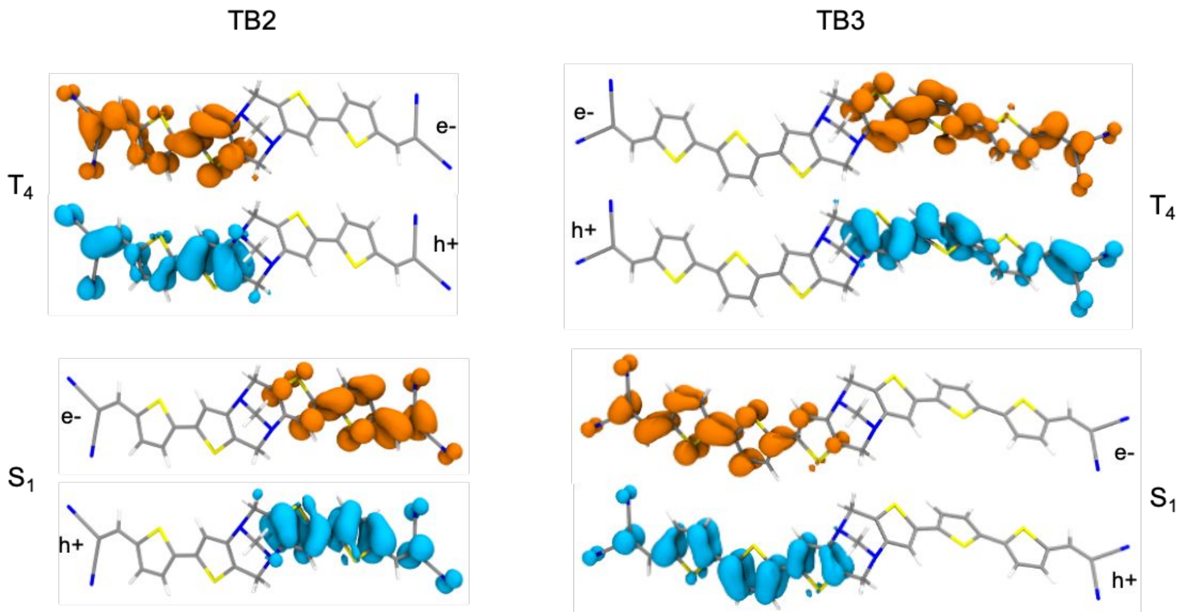


**Figure S21.** Electron/hole pair densities (orange/blue) for the $S_1$ and $T_4$ states of **TB2** and **TB3** at their respective relaxed $S_1$ geometries.

## Structural change upon excited state relaxation

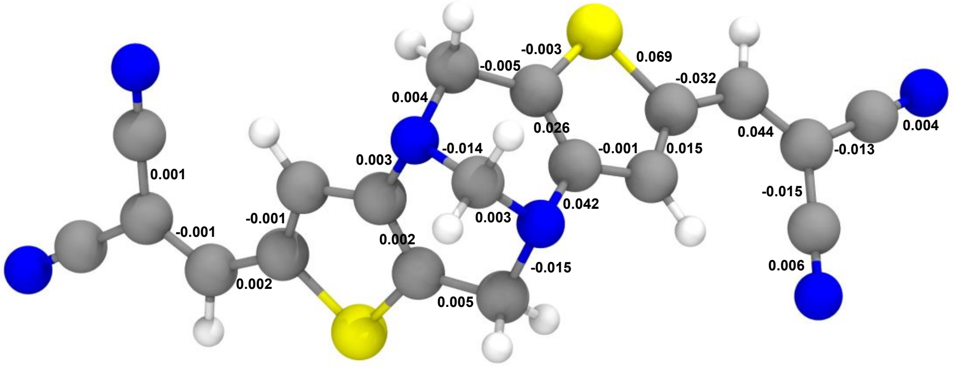


**Figure S22.** Changes in the bond lengths (in Å) at the relaxed $S_1$ geometry of TB1 with respect to its ground state equilibrium structure.

The change in bond lengths is accompanied by the planarization of the DCVT unit upon which the excitation is located ($\theta_6$=180°, cf. Figure S1).

## Molecular orbitals at the relaxed $S_1$ geometry

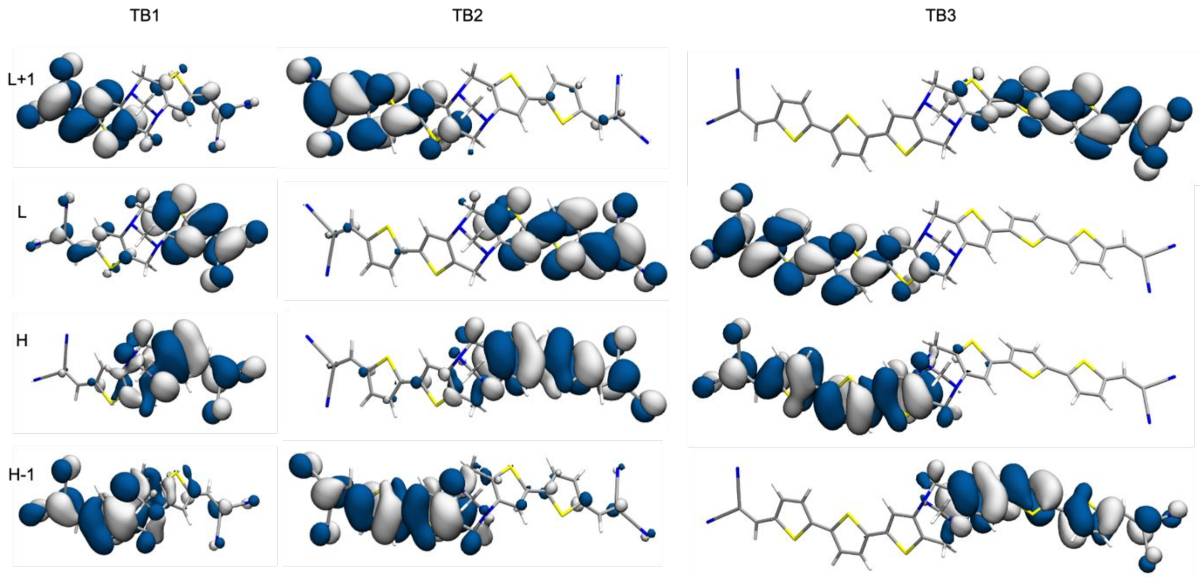


**Figure S23.** Molecular orbitals (HOMO-1, HOMO, LUMO and LUMO+1) of **TB1**, **TB2** and **TB3** at their respective $S_1$ geometry.

## TDDFT data ($S_1$ geometry)

**Table S4.** Vertical transition energies (in eV), oscillator strengths (in parentheses) and orbital contributions of **TB1**, **TB2** and **TB3** at their respective $S_1$ geometry calculated at the TDDFT level, and experimental values corresponding to the emission.

| | exp | TDDFT | contributions |
|---|---|---|---|
| TB1 | 2.39 | 3.11 (0.42) | 93% H→L |
| TB2 | 2.28 | 2.75 (1.33) | 91% H→L |
| TB3 | 2.04 | 2.45 (1.79) | 92% H→L |

**Table S5**. TDDFT energy differences ($\Delta E$ in eV) between pairs of singlet and triplet states of monomer **DCVT2** and **TB2** evaluated at their respective relaxed $S_1$ geometry and associated spin-orbit coupling constants (SOC).

| | **TB2** | | **DCVT2** | |
|---|---|---|---|---|
| | $\Delta E(T_n-S_1)$ | SOC ($cm^{-1}$) | $\Delta E(T_n-S_1)$ | SOC ($cm^{-1}$) |
| $T_1$ | -1.79 | 0.3 | -1.90 | 0.0 |
| $T_2$ | -1.15 | 0.1 | -0.34 | 0.0 |
| $T_3$ | -0.30 | 0.2 | 0.58 | 0.3 |
| $T_4$ | -0.01 | 0.6 | 0.84 | 0.3 |
| $T_5$ | 0.49 | 0.9 | 0.92 | 0.3 |
| $T_6$ | 0.76 | 0.9 | 1.21 | 0.4 |

## e-h correlation plots ($S_1$ geometry)

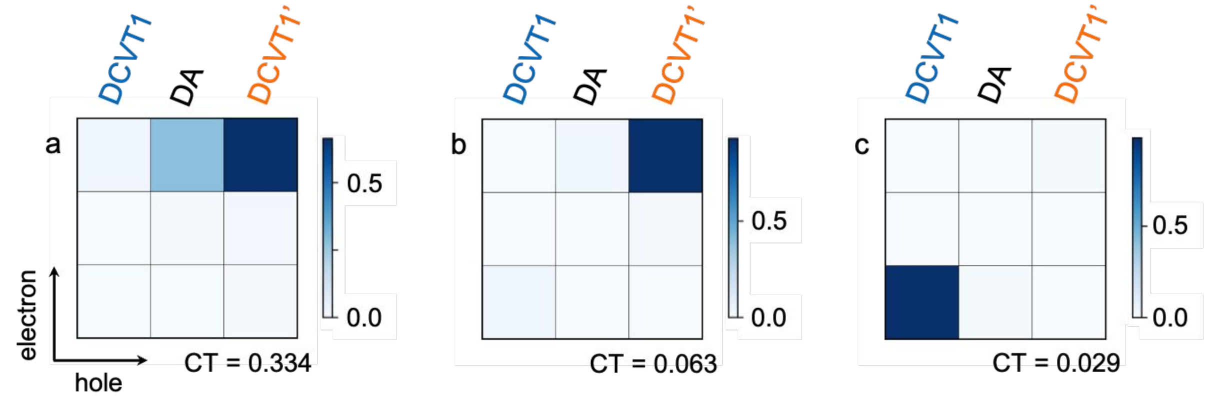


**Figure S24.** Electron-hole correlation plots for the $S_1$ state of **TB1** (a), **TB2** (b) and **TB3** (c) at their respective relaxed $S_1$ geometries, considering DA as central fragment (without CC units of thiophene). Results for **TB3** were obtained at the CAM-B3LYP/6-311G(d,p) level.

## Solvatochromic effects

**Table S6.** TDDFT energy differences ($\Delta E$ in eV), oscillator strength ($f$) and permanent dipole moment (DM in Debye) of the lowest excited singlet ($S_1$) of **TB1** at the ground (left) and excited (right) state geometries in toluene and THF computed at the CAM-B3LYP/6-31G(d,p) level. Ground state dipole moments in parenthesis.

| | $S_0$ geometry | | | $S_1$ geometry | | |
|---|---|---|---|---|---|---|
| solvent | $\Delta E$ | $f$ | DM | $\Delta E$ | $f$ | DM |
| toluene | 3.41 | 1.12 | 10.01 (6.05) | 2.92 | 0.59 | 12.95 (6.51) |
| THF | 3.38 | 1.09 | 10.74 (6.55) | 2.89 | 0.57 | 14.00 (7.45) |

# 3. Transient electronic Absorption Spectroscopy

### Qualitative comparison of triplet population

To obtain a concrete value for triplet absorptivity is a difficult task which involves the use of suitable triplet probes. These probes require complementary ground absorbance spectrum to our materials. In addition, their triplet absorbance spectra must appear in different places in the spectrum. These problems are further enhanced with materials with different absorbance spectra like the TBs, which would require to use different probe which would establish further error in the measurement.
Therefore, we did a simple qualitative comparison assuming that the TBs triplet relative absorptivity would be similar to their relative ground state absorptivity to obtain a superficial estimation. Assuming this, we obtained the following graph indicating the following trend in terms of triplet population **TB3**>**TB2**>>>**TB1**.

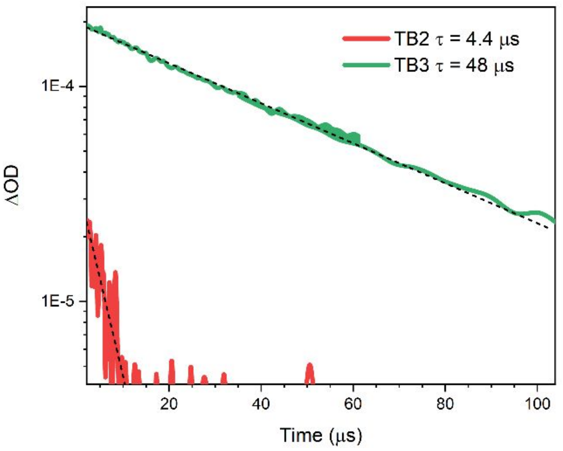


**Figure S25**. TA decays of the triplet population characterized by μs-TAS. **TB2** and **TB3** solutions were excited at 100 μJ/cm$^2$ at 450 and 550 nm, respectively. Fittings have been added as dashed lines to help the eye.

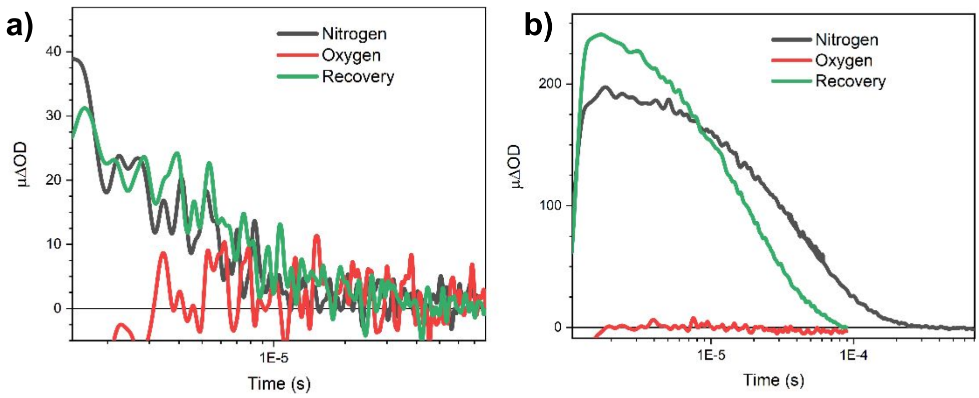


**Figure S26**. Oxygen dependence experiments for (a) **TB2** and (b) **TB3**. TA decays were recorded under nitrogen (black), oxygen (red) and back to nitrogen (green). **TB2** and **TB3** solutions were excited at 100 μJ/cm$^2$ at 450 and 550 nm, respectively.

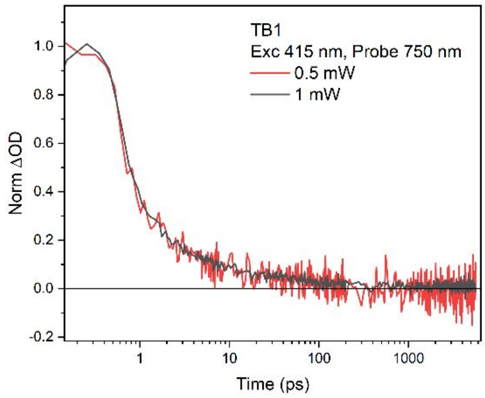


**Figure S27**. ps-TA decays of **TB1** solution probed at 750 excited at 415 nm at 0.5 (red) and 1 mW (black).

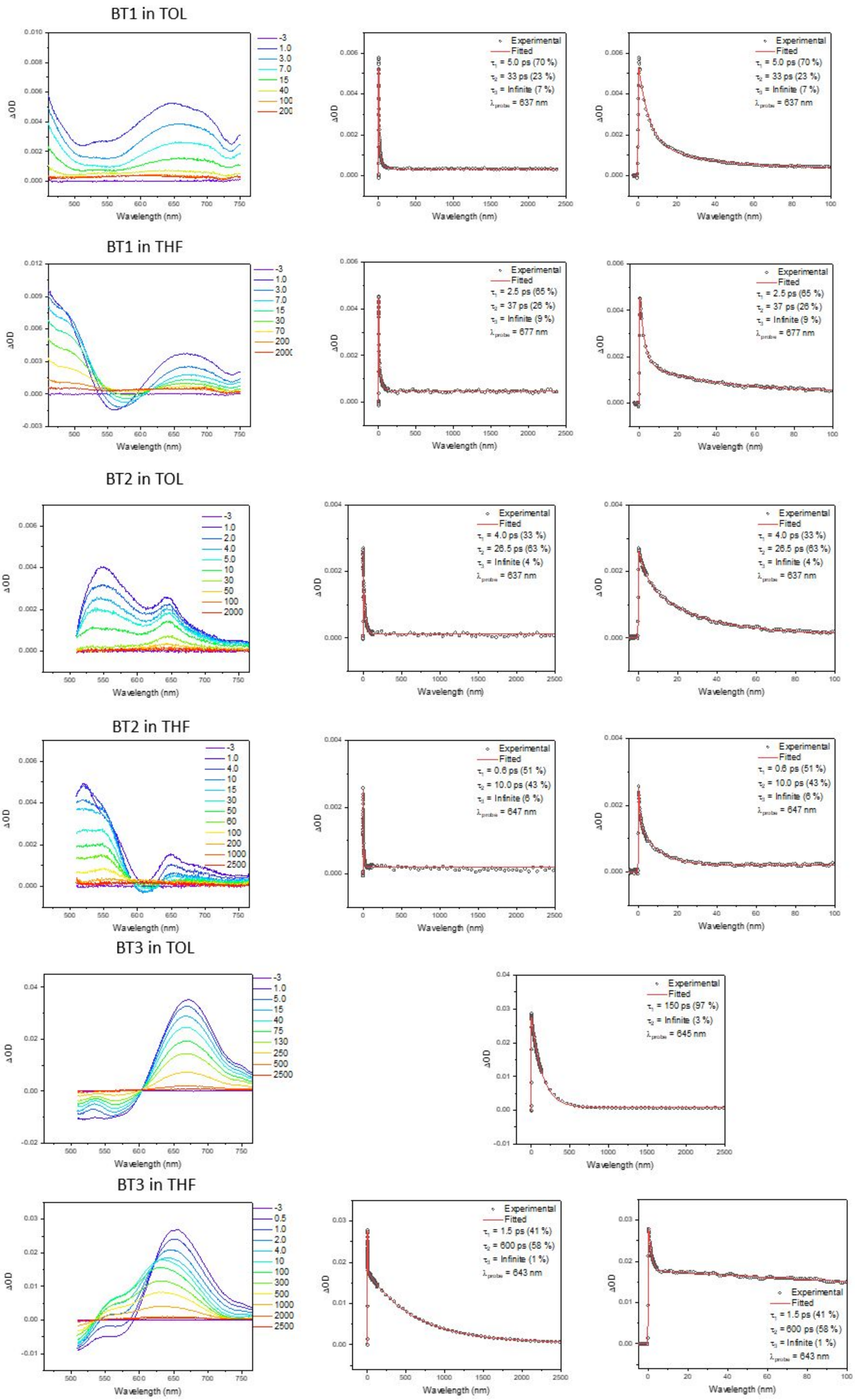


**Figure S28**. Pico-second transient absorption measurements for the three compounds under study carried out in: i) an apolar solvent as toluene and in a more polar solvent as THF. All at 298 K.

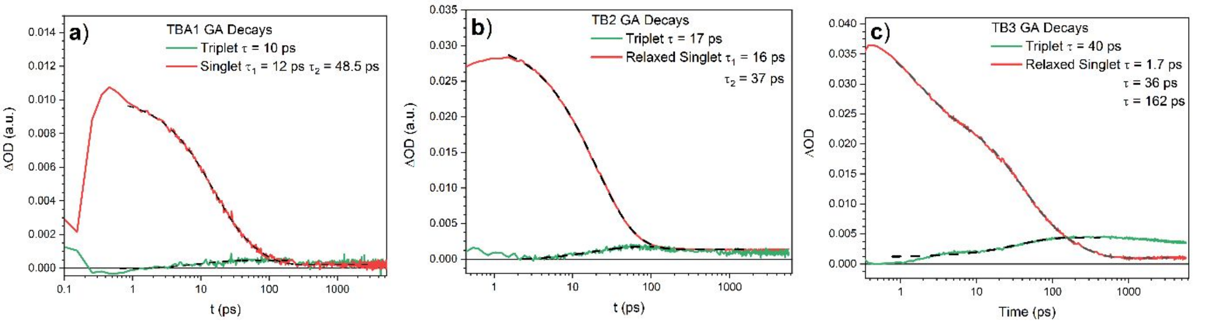


**Figure S29**. Global analysis decays obtained for (a) **TB1**, (b) **TB2** and (c) **TB3**. Fittings have been added as dashed lines to help the eye.

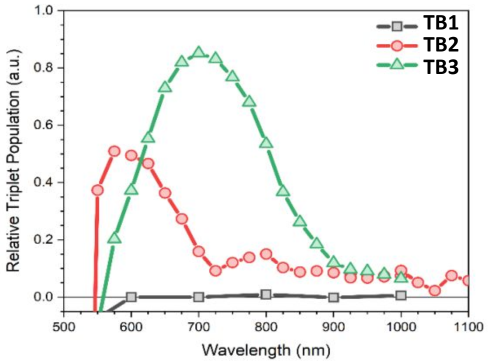


**Figure S30**. Relative population of triplet states for **TBn** materials using their ground state absorptivity and their µs-TAS spectra.

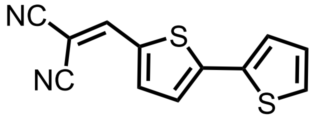


**Figure S31**. Chemical structure of **DCVT2** as the monomer reference of **TB2**. This was prepared as the non-brominated version of compound **4** in Scheme 1.

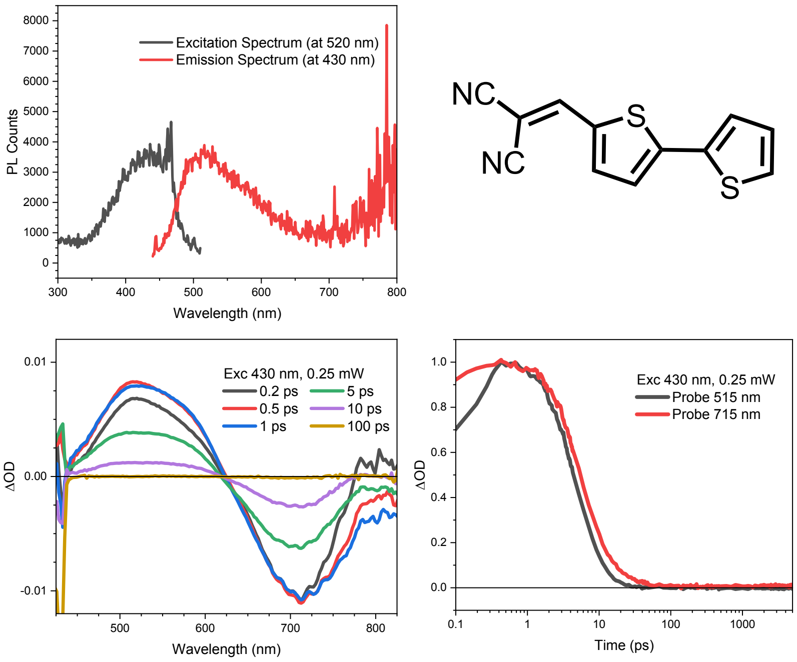


**Figure S32**. Top: Excitation spectrum of **DCVT2** (black line) in $CH_2Cl_2$ at 298 K compared with its emission spectrum (red line). Bottom, left: pico-second transient absorption spectra of **DCVT2** in $CH_2Cl_2$ at 298 K. Bottom, right: time evolution of the absorbance of the excited states with different probes at the two ESA bands with the same excitation at 430 nm.

## 4. Quantum chemical calculations for the *trans* conformers

**Table *trans* conformer.** Vertical transition energies (in eV), oscillator strengths (in parentheses) and orbital contributions of *trans* **TBn** at their respective ground state geometry calculated at the TDDFT level, and experimental values corresponding to the absorption maxima.

| | state | exp | TDDFT | contributions |
|---|---|---|---|---|
| TB1 | $S_1$ | 3.12 | 3.61 (0.62) | 70% H→L + 27% H-1→L+1 |
| trans | $S_2$ | / | 3.73 (0.18) | 53% H→L+1 + 40% H-1→L |
| TB2 | $S_1$ | 2.75 | 3.14 (1.48) | 56% H→L + 36% H-1→L+1 |
| trans | $S_2$ | / | 3.29 (0.36) | 50% H→L+1 + 43% H-1→L |
| TB3 | $S_1$ | 2.59 | 2.91 (1.85) | 48% H→L + 39% H-1→L+1 |
| trans | $S_2$ | / | 3.02 (0.82) | 45% H→L+1 + 42% H-1→L |

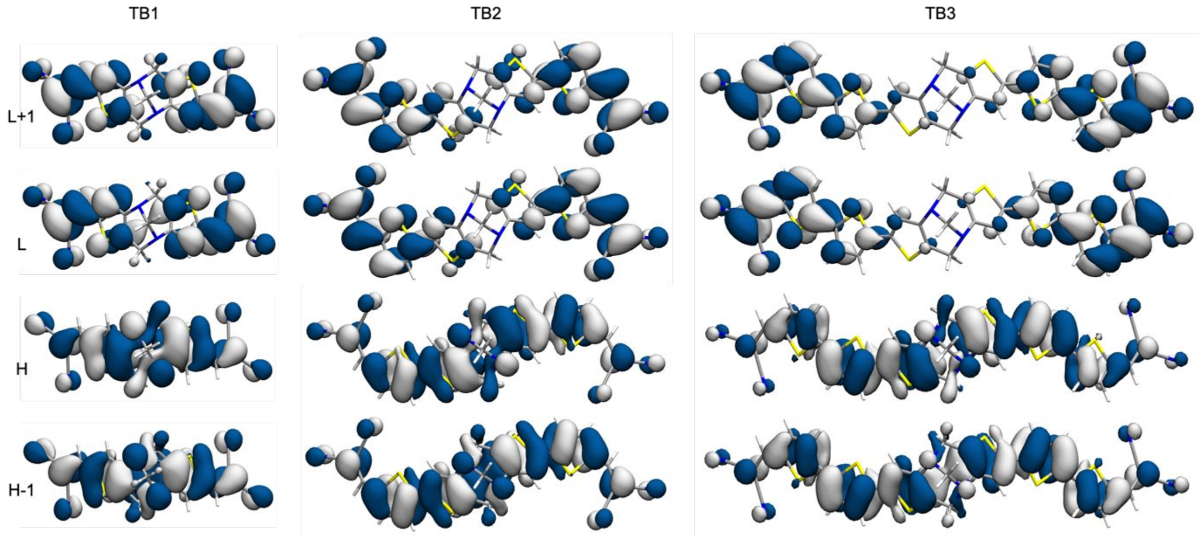


**Figure *trans* conformer.** Molecular orbitals (HOMO-1, HOMO, LUMO and LUMO+1) involved in the excitations to the lowest singlet excited states of *trans* **TBn** at their respective ground state geometry.

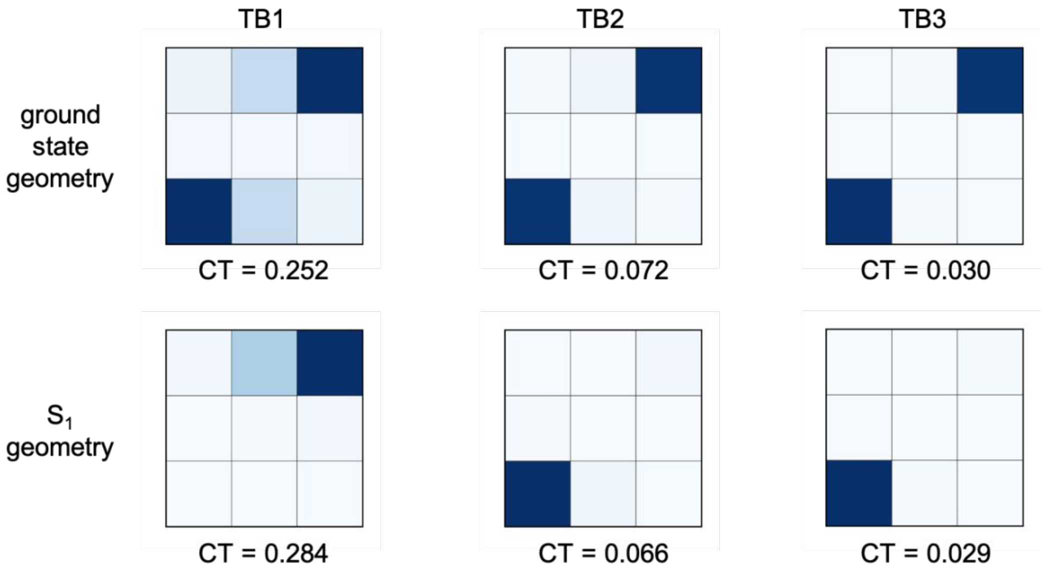


**Figure *trans* conformer.** Electron-hole correlation plots for the $S_1$ state of *trans* **TBn** at their respective ground state (top) and relaxed excited state (bottom) geometries (considering DA as the central fragment, without CC units of thiophenes). Results for **TB3** at the $S_1$ geometry were obtained at the CAM-B3LYP/6-311G(d,p) level.

**Table *trans* conformer.** Vertical transition energies (in eV), oscillator strengths (in parentheses) and orbital contributions of *trans* **TBn** at their respective $S_1$ geometry calculated at the TDDFT level, and experimental values corresponding to the emission.

| | exp | TDDFT | contributions |
|---|---|---|---|
| TB1 | 2.39 | 2.98 (0.27) | 93% H→L |
| TB2 | 2.28 | 2.74 (1.19) | 92% H→L |
| TB3 | 2.04 | 2.45 (1.65) | 92% H→L |

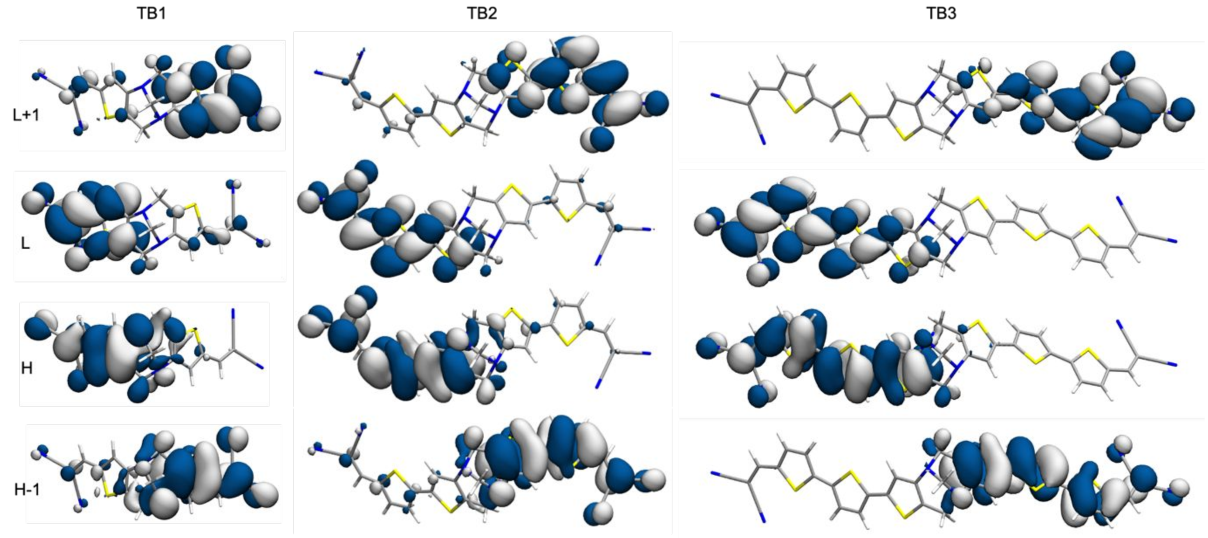


**Figure *trans* conformer.** Molecular orbitals (HOMO-1, HOMO, LUMO and LUMO+1) involved in the excitations to the lowest singlet excited states of *trans* **TBn** at their respective $S_1$ geometry.

**Table *trans* conformer.** TDDFT energy differences (ΔE in eV) between pairs of singlet triplet states of *trans* **TBn** together with the spin-orbit coupling constants (SOC), calculated at the relaxed $S_1$ geometry.

| | TB1 trans | | TB2 trans | | TB3 trans | |
|---|---|---|---|---|---|---|
| | $\Delta E(T_n-S_1)$ | SOC ($cm^{-1}$) | $\Delta E(T_n-S_1)$ | SOC ($cm^{-1}$) | $\Delta E(T_n-S_1)$ | SOC ($cm^{-1}$) |
| $T_1$ | -1.64 | 2.7 | -1.77 | 0.3 | -1.66 | 0.2 |
| $T_2$ | -1.03 | 0.5 | -1.13 | 0.1 | -0.93 | 0.1 |
| $T_3$ | -0.37 | 1.7 | -0.25 | 0.3 | -0.49 | 0.0 |
| $T_4$ | 0.24 | 2.8 | 0.04 | 0.6 | -0.13 | 0.1 |
| $T_5$ | 0.49 | 11.6 | 0.46 | 1.2 | 0.52 | 0.3 |
| $T_6$ | 0.76 | 0.5 | 0.76 | 1.3 | 0.72 | 0.6 |
| $T_7$ | 0.91 | 0.5 | 0.77 | 1.3 | 0.93 | 1.0 |
| $T_8$ | 1.06 | 11.7 | 0.89 | 0.6 | 1.01 | 0.7 |

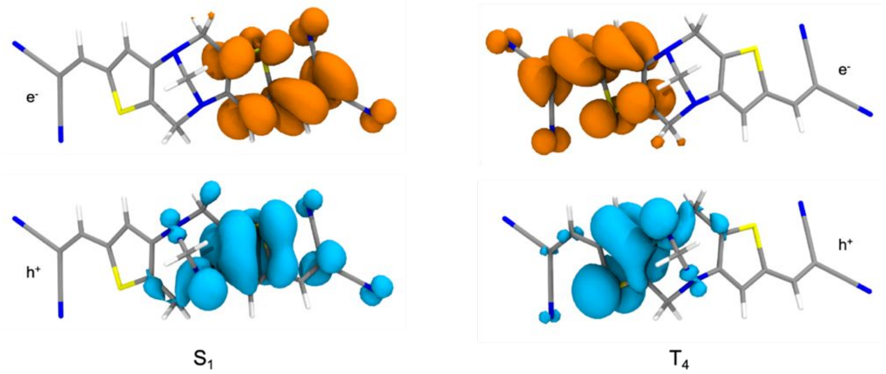


**Figure *trans* conformer.** Electron/hole pair densities (orange/blue) for the $S_1$ and $T_4$ states of *trans* **TB1** at the relaxed $S_1$ geometry.

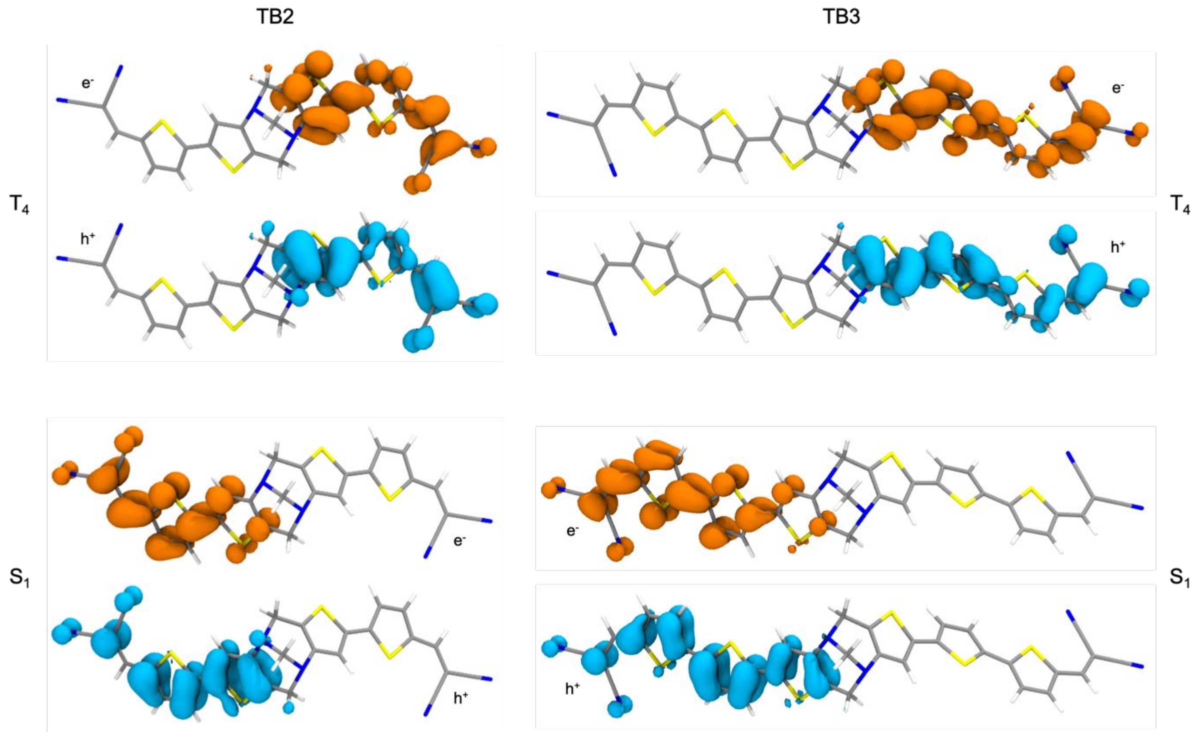


**Figure *trans* conformer.** Electron/hole pair densities (orange/blue) for the $S_1$ and $T_4$ states of *trans* **TB2** and **TB3** at their respective relaxed $S_1$ geometries.

## 5. References

1. Szłapa, A.; Kula, S.; Błaszkiewicz, U.; Grucela, M.; Schab-Balcerzak, E.; Filapek, M., Simple donor–π–acceptor derivatives exhibiting aggregation-induced emission characteristics for use as emitting layer in OLED. *Dyes and Pigments* **2016,** *129*, 80-89.
2. Raposo, M. M. M.; Fonseca, A. M. c. C.; Kirsch, G., Synthesis of donor–acceptor substituted oligothiophenes by Stille coupling. *Tetrahedron* **2004,** *60* (18), 4071-4078.
3. Kobayashi, T.; Moriwaki, T.; Tsubakiyama, M.; Yoshida, S., Synthesis and functionalization of thiophene congeners of Tröger's base. *Journal of the Chemical Society, Perkin Transactions 1* **2002,** (17), 1963-1967.
4. Martínez, Á. M.; Rodríguez, N.; Arrayás, R. G.; Carretero, J. C., Copper-catalyzed ortho-C–H amination of protected anilines with secondary amines. *Chemical Communications* **2014,** *50* (21), 2801-2803.
5. Plasser, F., TheoDORE: A toolbox for a detailed and automated analysis of electronic excited state computations. *The Journal of Chemical Physics* **2020,** *152* (8), 084108.
6. G. W. S. M. J. T. Frisch, H. B. S., G. E.; Robb, M. A.; Cheeseman, J. R.; Scalmani, G.; Barone, V.; Petersson, G. A.; Nakatsuji, H.; Li, X.; Caricato, M.; Marenich, A. V.; Bloino, J.; Janesko, B. G.; Gomperts, R.; Mennucci, B.; Hratchian, H. P.; Ortiz, J. V.; Izmaylov, A. F.; Sonnenberg, J. L.; Williams-Young, D.; Ding, F.; Lipparini, F.; Egidi, F.; Goings, J.; Peng, B.; Petrone, A.; Henderson, T.; Ranasinghe, D.; Zakrzewski, V. G.; Gao, J.; Rega, N.; Zheng, G.; Liang, W.; Hada, M.; Ehara, M.; Toyota, K.; Fukuda, R.; Hasegawa, J.; Ishida, M.; Nakajima, T.; Honda, Y.; Kitao, O.; Nakai, H.; Vreven, T.; Throssell, K.; Montgomery, J. A., Jr.; Peralta, J. E.; Ogliaro, F.; Bearpark, M. J.; Heyd, J. J.; Brothers, E. N.; Kudin, K. N.; Staroverov, V. N.; Keith, T. A.; Kobayashi, R.; Normand, J.; Raghavachari, K.; Rendell, A. P.; Burant, J. C.;

Iyengar, S. S.; Tomasi, J.; Cossi, M.; Millam, J. M.; Klene, M.; Adamo, C.; Cammi, R.; Ochterski, J. W.; Martin, R. L.; Morokuma, K.; Farkas, O.; Foresman, J. B.; Fox, D. J., Gaussian, Inc., Wallingford CT. **2016**.

7. Epifanovsky, E.; Gilbert, A. T. B.; Feng, X.; Lee, J.; Mao, Y.; Mardirossian, N.; Pokhilko, P.; White, A. F.; Coons, M. P.; Dempwolff, A. L.; Gan, Z.; Hait, D.; Horn, P. R.; Jacobson, L. D.; Kaliman, I.; Kussmann, J.; Lange, A. W.; Lao, K. U.; Levine, D. S.; Liu, J.; McKenzie, S. C.; Morrison, A. F.; Nanda, K. D.; Plasser, F.; Rehn, D. R.; Vidal, M. L.; You, Z.-Q.; Zhu, Y.; Alam, B.; Albrecht, B. J.; Aldossary, A.; Alguire, E.; Andersen, J. H.; Athavale, V.; Barton, D.; Begam, K.; Behn, A.; Bellonzi, N.; Bernard, Y. A.; Berquist, E. J.; Burton, H. G. A.; Carreras, A.; Carter-Fenk, K.; Chakraborty, R.; Chien, A. D.; Closser, K. D.; Cofer-Shabica, V.; Dasgupta, S.; de Wergifosse, M.; Deng, J.; Diedenhofen, M.; Do, H.; Ehlert, S.; Fang, P.-T.; Fatehi, S.; Feng, Q.; Friedhoff, T.; Gayvert, J.; Ge, Q.; Gidofalvi, G.; Goldey, M.; Gomes, J.; González-Espinoza, C. E.; Gulania, S.; Gunina, A. O.; Hanson-Heine, M. W. D.; Harbach, P. H. P.; Hauser, A.; Herbst, M. F.; Hernández Vera, M.; Hodecker, M.; Holden, Z. C.; Houck, S.; Huang, X.; Hui, K.; Huynh, B. C.; Ivanov, M.; Jász, Á.; Ji, H.; Jiang, H.; Kaduk, B.; Kähler, S.; Khistyaev, K.; Kim, J.; Kis, G.; Klunzinger, P.; Koczor-Benda, Z.; Koh, J. H.; Kosenkov, D.; Koulias, L.; Kowalczyk, T.; Krauter, C. M.; Kue, K.; Kunitsa, A.; Kus, T.; Ladjánszki, I.; Landau, A.; Lawler, K. V.; Lefrancois, D.; Lehtola, S.; Li, R. R.; Li, Y.-P.; Liang, J.; Liebenthal, M.; Lin, H.-H.; Lin, Y.-S.; Liu, F.; Liu, K.-Y.; Loipersberger, M.; Luenser, A.; Manjanath, A.; Manohar, P.; Mansoor, E.; Manzer, S. F.; Mao, S.-P.; Marenich, A. V.; Markovich, T.; Mason, S.; Maurer, S. A.; McLaughlin, P. F.; Menger, M. F. S. J.; Mewes, J.-M.; Mewes, S. A.; Morgante, P.; Mullinax, J. W.; Oosterbaan, K. J.; Paran, G.; Paul, A. C.; Paul, S. K.; Pavošević, F.; Pei, Z.; Prager, S.; Proynov, E. I.; Rák, Á.; Ramos-Cordoba, E.; Rana, B.; Rask, A. E.; Rettig, A.; Richard, R. M.; Rob, F.; Rossomme, E.; Scheele, T.; Scheurer, M.; Schneider, M.; Sergueev, N.; Sharada, S. M.; Skomorowski, W.; Small, D. W.; Stein, C. J.; Su, Y.-C.; Sundstrom, E. J.; Tao, Z.; Thirman, J.; Tornai, G. J.; Tsuchimochi, T.; Tubman, N. M.; Veccham, S. P.; Vydrov, O.; Wenzel, J.; Witte, J.; Yamada, A.; Yao, K.; Yeganeh, S.; Yost, S. R.; Zech, A.; Zhang, I. Y.; Zhang, X.; Zhang, Y.; Zuev, D.; Aspuru-Guzik, A.; Bell, A. T.; Besley, N. A.; Bravaya, K. B.; Brooks, B. R.; Casanova, D.; Chai, J.-D.; Coriani, S.; Cramer, C. J.; Cserey, G.; DePrince, A. E., III; DiStasio, R. A., Jr.; Dreuw, A.; Dunietz, B. D.; Furlani, T. R.; Goddard, W. A., III; Hammes-Schiffer, S.; Head-Gordon, T.; Hehre, W. J.; Hsu, C.-P.; Jagau, T.-C.; Jung, Y.; Klamt, A.; Kong, J.; Lambrecht, D. S.; Liang, W.; Mayhall, N. J.; McCurdy, C. W.; Neaton, J. B.; Ochsenfeld, C.; Parkhill, J. A.; Peverati, R.; Rassolov, V. A.; Shao, Y.; Slipchenko, L. V.; Stauch, T.; Steele, R. P.; Subotnik, J. E.; Thom, A. J. W.; Tkatchenko, A.; Truhlar, D. G.; Van Voorhis, T.; Wesolowski, T. A.; Whaley, K. B.; Woodcock, H. L., III; Zimmerman, P. M.; Faraji, S.; Gill, P. M. W.; Head-Gordon, M.; Herbert, J. M.; Krylov, A. I., Software for the frontiers of quantum chemistry: An overview of developments in the Q-Chem 5 package. *The Journal of Chemical Physics* **2021,** *155* (8).
